# Supplementary material for: Adjuvant rituximab, a potential treatment for the young patient with Graves’ hyperthyroidism (RiGD): study protocol for a single-arm, single-stage, phase II trial
Source: BMJ Open. 2019 Jan 21;9(1):e024705. doi: 10.1136/bmjopen-2018-024705 (PMC6347892; doi:10.1136/bmjopen-2018-024705)
Supplement: Supplementary file 1 [file bmjopen-2018-024705supp001.pdf]

PARTICIPANT INFORMATION SHEET FOR CHILDREN

Short title: Rituximab in Graves' Disease

Study Question?

Will a new medicine called Rituximab help to make the thyroid gland work normally.

Brief Summary

Current standard NHS treatment for patients with Graves' Disease	Treatment you will get if you take part in this study.
1. You will see your doctor around 10 times over 2 years.	1. You will see your doctor 15 times over 2 years.
2. You will take anti-thyroid medicine for 2 years.<br>(Once patients stop taking this medicine the disease will usually come back in 3 out of 4 patients)	2. You will take anti-thyroid medicine for 1 year and then be followed up in clinic for the next year.
3. If the disease comes back the options include surgery (removing your thyroid gland) or Radioiodine treatment (radioactive iodine that is taken up by the thyroid and destroys it) or returning to anti-thyroid medicine.<br>After surgery or radioiodine treatment, patients usually take thyroid hormone replacement therapy for life.	3. You will get one dose of Rituximab treatment using a tube in a vein with a drip attached. This treatment may help to reduce the chances of the disease coming back when you stop the anti-thyroid medicine after 1 year.<br>If your thyroid gland is still not working normally after the first year of treatment with us, we will be able to detect this. If this happens we will restart your anti-thyroid medicine.<br>After the first year of treatment is finished, it is possible that your thyroid problem might come back. If this happens please let us know straight away and we will restart anti-thyroid medicine so you do not feel poorly.

What is Graves' Disease?

You have been found to have Graves' Disease. In Graves' disease the thyroid gland in the neck makes too much thyroid hormone. This can make you feel unwell.

Why me?

You have been picked because you have Graves' Disease. You can help us find out if a new medicine called Rituximab will help to make the thyroid gland work

normally. The Rituximab medicine will be given together with a one year course of the normal treatment for this condition. Unfortunately the normal treatment (anti-thyroid drug – usually taken as tablets that you swallow) does not usually make the thyroid gland work normally by itself.
We are asking 27 young people between 12 and 20 years old to take part in this study.

Do I have to take part?
No, you do not. It is up to you. We would first like you to read this information sheet with your mum, dad or carer and if you would like to take part we would then like you to confirm this by writing your name on a form.
We will also ask your mum, dad or carer to write down that they are happy for you to take part as well.
Remember, you can still change your mind later. If you don't want to take part just tell us.

What will happen?
As you have Graves' Disease, you would normally see your doctor about 10 times over 2 years. If you take part in the study we would like to see you 15 times over the next 2 years. If you have Graves' disease you would normally get a blood sample taken when you see your doctor. We would like to take a little extra blood to make sure that you are OK and to see if the new medicine is working and to do new tests to see how your immune system is working. If you are a girl, we will need to take some of your urine, 2 times during the study. We check your urine to make sure you are not pregnant. This is something we have to do in every female that takes part, irrespective of how old they are. Both the girls and boys that decide to take part in this study will need to use contraception if there is chance that you might get pregnant or have a baby. This is because it might not be good for the baby if you get pregnant in the 12 months after receiving the Rituximab medicine. Information on the different methods of contraception available can be found on the NHS webpage http://www.nhs.uk/Conditions/contraception-guide/Pages/what-is-contraception.aspx or call the national sexual health line on 0300 123 7123. You can also talk to the study team if you have any questions.

What happens when the study stops?
We will collect the information from the young people taking part and we will then decide if we need to have a bigger study. A bigger study will help to tell us for certain if Rituximab should be used in Graves' Disease in the future.

What if there is a problem?
Your mum, dad or carer will be able to talk to someone who will be able to tell them what they need to do about it.

What if I don't want to do the research anymore?
Just tell your mum, dad, carer or doctor. They will not be cross with you. You will still be looked after when you are visiting hospital.

What if I wish to complain about the study?
If you have a concern about any aspect of this study or if you want to complain, you, or your mum, dad or carer can talk to your doctor ____ or nurse ____ at this hospital.

Will anyone else know I'm doing this study?
The people in our research team will know you are taking part. The doctor looking after you while you are in hospital will also know and we will also inform your family doctor or GP. No one else will know because we will not use your name or address.

What happens to what the researchers find out?
When we collect your information we will make sure it is stored in a safe place and only the people doing the research study can look at it.
We will use the information to work out how best to treat Graves' Disease. The Newcastle Clinical Trials Unit would like to receive a copy of your consent form for safety purposes. This will be destroyed once it has been reviewed.

How can I find out more about this study?
Your mum, dad, carer or other grown-up you trust may be able to answer some of your questions. The doctors and nurses looking after you can also help you all to find out more about the study.

Rituximab Treatment
Your nurse or doctor will give you Rituximab once using a tube in a vein with a drip attached. Local anaesthetic cream will be offered before the tube is put in place or before any blood tests so that it doesn't hurt. The drip will have a pump at the other end that will give you Rituximab over about 3 to 4 hours.

What could it do to me? Is it definitely safe?
Rituximab has been used to treat other diseases in lots of young and old people for the past 18 years. Half of the patients may feel a bit hot or cold, or sick or itchy for a short time but we will give you medicine to help prevent this. If you do feel poorly during the treatment then we will stop giving you Rituximab for a few minutes. When you feel better again we will give you Rituximab more slowly. You will be checked carefully as you get Rituximab to make sure you are feeling ok. There is a very small possibility that you might need antibiotic medicine to help you fight infections at some point in the next year. Although there is no guarantee that Rituximab and Anti Thyroid Drug (ATD) treatment will work for you, We need to do this study to work out how likely it is that this approach will benefit young people.

Thank you for taking time to read this –

Please ask any questions if you need to.
