# Supplementary material for: Adjuvant rituximab, a potential treatment for the young patient with Graves’ hyperthyroidism (RiGD): study protocol for a single-arm, single-stage, phase II trial
Source: BMJ Open. 2019 Jan 21;9(1):e024705. doi: 10.1136/bmjopen-2018-024705 (PMC6347892; doi:10.1136/bmjopen-2018-024705)
Supplement: Supplementary file 2 [file bmjopen-2018-024705supp002.pdf]

To be printed on local hospital headed paper

Adjuvant rituximab – a potential treatment for the young patient with Graves' hyperthyroidism.

Short title: Rituximab in Graves' Disease

PATIENT INFORMATION LEAFLET

Invitation

You are being invited to take part in a research study. Please read the following information to help you decide if you want to take part. We would like you to understand why we are doing this research and what it means for you. You do not need to make a decision straight away, so please feel free to talk to others about the study if you wish. Please ask us if there is anything that is not clear or if you want to know more.

Please remember that you do not have to take part and your normal healthcare will not be affected in any way, whatever you decide.

(Brief summary to see if you want to continue to read)

- You have been asked to take part because you have been diagnosed with Graves' hyperthyroidism (also known as Graves' disease), which means you have an overactive thyroid gland. We are looking at a possible new treatment for Graves' disease using a medicine called Rituximab.

- The current standard treatment for Graves' disease involves;
  - Taking Anti Thyroid Drugs (ATD) for around 2 years and seeing your doctor for a check-up about 10 times over the same period of time. The Graves' disease will come back once you have stopped taking ATD in about 3 out of every 4 patients.
  - If ATD treatment does not work and the Graves' disease comes back then the other current treatment options (apart from returning to the ATD once again) are;

    1.) Surgery (removing your thyroid gland)

    2.) Radioiodine treatment. (Radioactive iodine is taken up by the thyroid, and destroys the cells in the thyroid gland).

- After surgery or radioiodine treatment, patients usually need to take thyroid hormone replacement for the rest of their life. Thyroid hormone replacement medicine is needed to replace the hormones which are missing after removal of the thyroid gland by surgery or after destruction by radioiodine treatment.

Why is a trial needed?

A trial is needed because the potential new treatment of Graves' disease that uses a combination of Rituximab and a short course of anti-thyroid drugs has not been studied before. Rituximab is given into a vein with a drip (an intravenous infusion) on one occasion and the anti-thyroid drugs are the tablets that are normally taken in this condition by mouth (orally). We do not know if this new treatment combination is more likely to restore your thyroid gland function to normal. The idea behind the study is that Rituximab might slow down or stop the the body's immune system from making your thyroid gland over-active. We would like to find out if the single dose of Rituximab, when taken together with a 1 year course of anti-thyroid drug, will increase the likelihood of the thyroid gland functioning normally when anti-thyroid drug treatment is stopped. This trial will recruit 27 young people, aged 12-20 years, who have recently been diagnosed with Graves' disease and who have only been on anti-thyroid drugs for 6 weeks at the most.

Standard or usual NHS care would mean that you see your thyroid doctor about 10 times over 2 years. If you take part in this trial we would like to see you 15 times over 2 years.

Please continue to read the following information to see if you would like to take part.

What is Rituximab?

Patients with Graves' Disease have too many abnormal B cells that are part of the body's immune system. Rituximab targets abnormal and normal B-cells. Once Rituximab treatment is over the normal B-cells will recover but we hope that the abnormal ones will not be replaced.

What would taking part involve?

<u>In total we will need to see you 15 times in 2 years.</u>

Introductory visit (Total time approx. 30 minutes)

We will discuss the study in full and you can ask any questions you might have about your diagnosis. You would then go away and think about it for a few days.

Screening and Consent visit (total time 1 hour)

We will check that you understand the study and are happy to take part. We will then ask you to sign a consent form and we will take a blood sample (less than 1 teaspoon in amount) to check for a viral infection of the liver (hepatitis) and also (only for female patients) to undertake a pregnancy test. This is something we have to do for every female that takes part, irrespective of what age they are. If either of these tests are positive we will let you know. This means that you would not be eligible to take part in the study. You would then return to standard NHS care. Some units may routinely screen for other infections before administering Rituximab.

Rituximab Treatment visit (Total time 6 hours).

This visit will only happen if the screening visit bloods confirm that there is no evidence of hepatitis infection and that you are not pregnant (female patients). You will then have a thin plastic cannula (tube) inserted into your arm vein and 10mls of blood (less than 3 teaspoons) will be taken. Local anaesthetic cream will be offered before the tube is put in place or before any blood tests. A member of the study team will then give you Rituximab into a vein with a drip (an intravenous infusion). They will give the Rituximab treatment using a pump, which will give you the medicine over a 3 to 4 hours. You will be monitored in hospital for a further 2 hours following the treatment before you can go home. After this visit and subsequent visits a member of the team will call you to tell you what dose of anti-thyroid drugs you need to take.

For the next 12 months (Visit times 30 minutes approx.)

During the first 12 months of the study you will be seen in hospital to review the dose of your anti-thyroid drug. We will check to see how you are feeling and record if you have noticed any side effects. We will then take up to 8 mls of blood, less than 2 teaspoons of blood to make sure that you are OK. After each

visit a member of the study team will call you to tell you what dose of anti-thyroid drug you need to take.

1 year after Rituximab Treatment (Visit time 30 minutes approx.).
You will be asked to stop taking anti-thyroid drugs at this visit. We will check to see how you are feeling and record if you have noticed any side effects. We will need to take 8mls of blood (less than 3 teaspoons) to make sure you are OK and for an investigation into newly discovered markers of the body's immune system works. Female patients will need to give a urine sample to rule out pregnancy.

The final 12 months (Each visit time lasts half an hour approximately.)
When you come to the hospital we will check to see what medications you are taking. We will check to see how you are feeling. If the hyperthyroidism returns then we may detect this before you develop symptoms. If you start to feel as though the hyperthyroidism is coming back between clinic visits then you can let us know straight away so you don't become poorly. We will need to take 12mls of blood (less than 3 teaspoons) to make sure that you are OK and to see if the Graves' disease has returned. Once the 2 years are over you will then return to normal NHS endocrinology care.

SUMMARY of main study visits

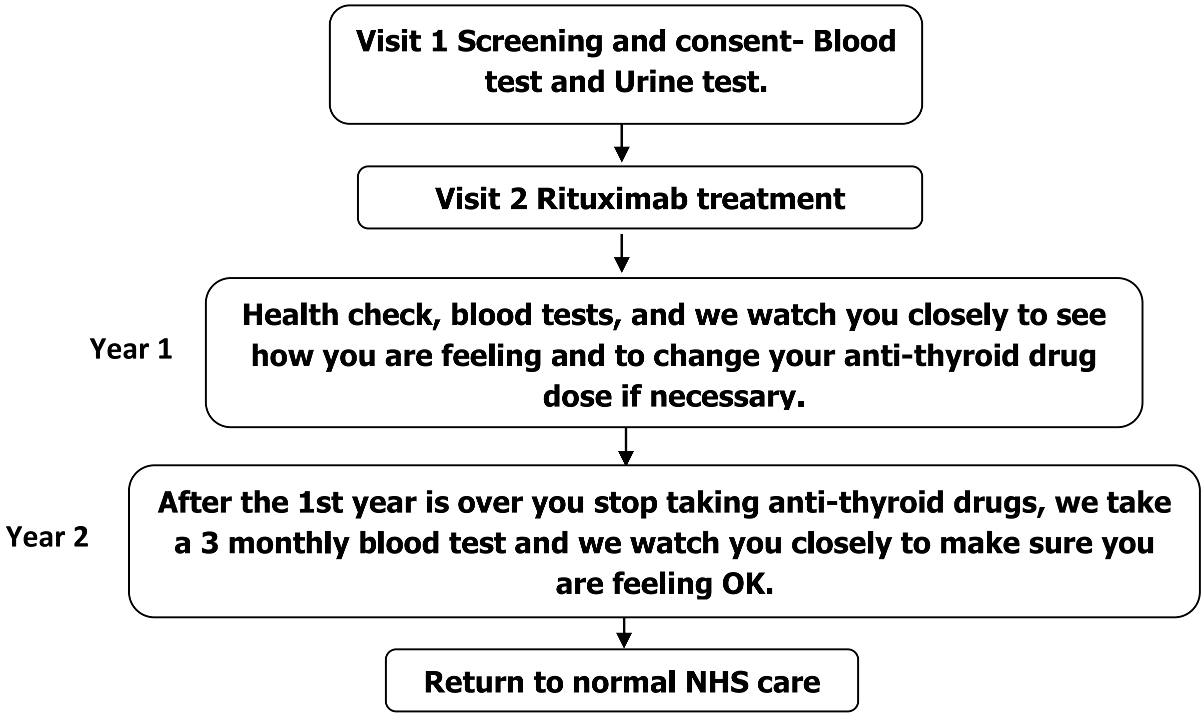

What is it like to receive Rituximab?

Rituximab has been in use for over 18 years, with more than a million people treated world-wide – including many young people. It has been used successfully in many people with other autoimmune conditions (Graves' disease is also an autoimmune condition). You will receive one dose of the Rituximab. Around half of the people receiving a Rituximab infusion will feel some effects from having the drug. These can include, feeling hot or cold, shivering, feeling sick, or itchiness. We use other medication (such as paracetamol) to prevent this but if it does happen then the treatment will be stopped for a few minutes, until you feel better, and then restarted at a slower rate. You will be monitored carefully during the treatment and watched carefully if you feel poorly.

What happens after the treatment?

Following the treatment, you will go home with your anti-thyroid tablets. We advise for the first 6 months following treatment that patients do not receive any live vaccines. For the first 12 months we will want to keep a close eye on you to check you are well and, if necessary, to adjust the dose of the anti-thyroid drug. We expect that your dose of anti-thyroid tablets will be reduced during the first year of the study. For the final 12 months of the study we will see you every 3 months to check you are well and to see if the Graves' disease has returned.

What do I do next if I am considering taking part in the study?

You may have further questions to ask, and you may wish to discuss it with family members, friends, your own family doctor or hospital specialist. Please ask the study team anything you like about this trial. Remember that if you do agree to participate, you can decide not to continue at any stage and that your treatment will not be affected in any way, whatever you decide about the study. Take your time, and tell your hospital consultant if you wish to go ahead.

RiGD

To be printed on local hospital headed paper

Your local contact people for the study are:

Contact Details of local PI:	Contact details of local Research Nurse:
Name:	Name:
Address:	Address:
Phone:	Phone:
Email:	Email:

What are the possible disadvantages and risks of taking part?

There is no guarantee that Rituximab and ATD treatment will work for you. We need to do this study to work out how likely it is that this approach will benefit patients.

As Rituximab is acting to alter your immune system, there is a small risk (1 in 50) that you might develop a serious infection, such as pneumonia, after the infusion. If this happens to you then you may have to be hospitalised to receive antibiotic treatment. However, most people have Rituximab without any infection occurring as a result.

You will be asked to come to the research unit 15 times during the 2 year study. This is a considerable commitment in terms of your time and you will need to think carefully about whether you can fit in this number of visits.

Can I take part if I (or my partner) am pregnant or are planning on becoming pregnant?

We cannot give Rituximab to any females that are pregnant or planning on becoming pregnant during the study, because of the unknown risks to unborn babies. We also cannot give Rituximab to any males that are planning a pregnancy with their partner during the study.

All males and females participating in the study <u>must</u> agree to use effective forms of contraception for at least 12 months following the Rituximab treatment,

(The study team will discuss effective forms of contraception with you.) Information on the different methods of contraception available can be found on the NHS webpage http://www.nhs.uk/Conditions/contraception-guide/Pages/what-is-contraception.aspx or call the national sexual health line on 0300 123 7123. Rituximab might stay in your body for up to 12 months after the infusion.

If you or your partner becomes pregnant during the study you must tell the research team immediately.

Will I be paid for being in the trial?

No, you will not be paid for being in the trial although we will reimburse all travel expenses.

What if there is a problem?

If you have a concern about any aspect of the study please contact your local doctor (see contact details above) or the doctor running this study, Dr Tim Cheetham (0191 282 9562 or ask for Dr Cheetham to be air-called via the Royal Victoria Infirmary Switchboard on 0191 233 6161) to discuss your concerns. If you are still unhappy and wish to complain formally and confidentially you can do this through the NHS complaints Procedure by speaking to a member of the PALS (Patient Advice and Liaison Service) on 0800 0320 202 or <site to localise with phone number and email address>

What will happen if I don't want to carry on with the study?

You can stop taking part in the study at any time and for any reason, without having to tell us why. We will keep any information and samples that you have given us so far for research. By signing the consent form you agree to this. If you do withdraw from the study, you will still need to be seen on a regular basis by a hospital team who are familiar with the normal management of Graves' disease.

Will my GP be told about my involvement in this study?

If you decide to take part in this study we will inform your GP. It will also be noted in your hospital medical records.

What will happen to any samples that I give?

You will have given us 15 blood samples for the study (normal NHS care would involve about 10). The serum from your blood for 3 of these samples will be sent to a laboratory at Newcastle upon Tyne. The team there will use the blood samples to look at the level of antibodies that cause Graves' disease and the levels of thyroid hormone and for an investigation into newly discovered markers of the way the body's immune system works. Two samples will be stored for about 5 years just in case we need to test for other antibodies in future.

Will my personal details be kept confidential?

All personal details will be kept confidential. The study data in your medical notes will be looked at by people directly involved in the study, as well as by people who are monitoring and auditing the study. This may include the Newcastle Clinical Trials Unit, regulatory authorities or the hospital to make sure the study is being run correctly. We will need to take your contact details to get in touch with you by telephone or via email to give you your new ATD dose after study visits 1-9. We may also need to contact you regarding long term thyroid status and longer term well-being. Any personal information will be held securely and safely for the time of the study at your hospital. When the study ends this information needs to be kept (archived) for at least 5 years. This allows any queries about the conduct of the study to be resolved. This archived information will be kept very securely by the hospital to protect you. Any data collected during the study held by the central study team at Newcastle will be anonymised and will only have your study ID included. The Newcastle Clinical Trials Unit would like to receive a copy of your consent form for safety purposes. This will be destroyed once it has been reviewed.

What will happen to the results of the research study?

The results of the study will be published in scientific journals. You will not be named in any publication. We would also like to send you a newsletter with a summary of our results. Please let the research team know if you do not want to receive the newsletter.

Who is organising and funding the research?

This study has been funded by the Medical Research Council, United Kingdom. This study is sponsored and indemnified by the Newcastle Upon Tyne NHS

Foundation Trust and Newcastle University. The Newcastle Clinical Trials Unit is organising the study.

Who has reviewed the study?

All research in the NHS is looked by an independent group of people called a NHS Research Ethics Committee (REC). This is to protect your interests. This study has been reviewed and given a favourable opinion by North East – Tyne & Wear South Research Ethics Committee and the NHS Health Research Authority.

What if relevant new information becomes available?

The study team will ensure the patients are receiving the most appropriate and up to date medical care they require.

How have patients and the public been involved in this study?

The Young Persons Advisory Group (YPAG) based in Newcastle upon Tyne have reviewed this Patient Information Leaflet and consent form. The Founding Director and Secretary to the Trustees of the British Thyroid Foundation has also reviewed this Patient Information Leaflet and consent form.

Thank you for taking time to read this leaflet.
