# Supplementary material for: Adjuvant rituximab, a potential treatment for the young patient with Graves’ hyperthyroidism (RiGD): study protocol for a single-arm, single-stage, phase II trial
Source: BMJ Open. 2019 Jan 21;9(1):e024705. doi: 10.1136/bmjopen-2018-024705 (PMC6347892; doi:10.1136/bmjopen-2018-024705)
Supplement: Supplementary file 3 [file bmjopen-2018-024705supp003.pdf]

To be printed on Hospital headed paper

Young Person aged (12-15 years) Participant Assent Form v4.0

Adjuvant rituximab – a potential treatment for the young patient with Graves' hyperthyroidism.

Short title: Rituximab in Graves' Disease

Centre Number: ___________________

Participant Number: _________________

		Please INITIAL the boxes if you agree:
1	I have read and understood the Participant Information Sheet version __________ dated ___________.	
2	I have had the chance to ask questions about the study and am happy with the answers given.	
3	I understand that I will need to give a blood sample when I come to hospital.	
4	I understand that some of the blood I give for this study will be used for new Exploratory analyses tests	
5	I understand that if I do not have to take part in the RiGD study, I know that I can stop at any time and I do not need to give a reason.	
6	I agree to take part in this study.	
7	I would like a copy or summary of the results to be sent to me when the study has finished.	
8	I agree to using the forms of contraception that have been explained to me if I am sexually active.	
9	I agree for a copy of this consent form to be sent securely to the Newcastle Clinical Trials Unit for safety purposes.	

To be answered by Female Participants only

10	I understand that I will need to provide 2 urine samples during the study to make sure that I am not pregnant. I understand that this is for safety purposes.	

**Name of Child**	**Child to write Name here**	**Date**
**Name of person taking consent**	**Signature**	**Date**

When completed 1 copy for participant, 1 for researcher site file, 1 (original) to be kept in medical notes and 1 copy to send securely to Newcastle Clinical Trials Unit
