# Supplementary material for: Adjuvant rituximab, a potential treatment for the young patient with Graves’ hyperthyroidism (RiGD): study protocol for a single-arm, single-stage, phase II trial
Source: BMJ Open. 2019 Jan 21;9(1):e024705. doi: 10.1136/bmjopen-2018-024705 (PMC6347892; doi:10.1136/bmjopen-2018-024705)
Supplement: Supplementary file 4 [file bmjopen-2018-024705supp004.pdf]

RiGD

To be printed on Hospital headed paper

Adjuvant rituximab – a potential treatment for the young patient with Graves' hyperthyroidism. (16yrs +)

Short title: Rituximab in Graves' Disease

Participant Consent Form V4.0

Centre Number: ___________________ **Participant Number:** _____________

Please INITIAL the boxes if you agree:

1	I have read and understood the Participant Information Sheet version _____ dated __________. I have had the opportunity to consider the information, to ask questions and am happy with the answers given.	
2	I understand that my participation is voluntary and that I am free to withdraw at any time without giving any reason, without my medical care or legal rights being affected.	
3	I consent to the collection of blood samples as part of this study. I understand that my samples will not be identifiable to me except to the study doctor and the direct research team.	
4	I understand that if I do withdraw and if I have already provided any of the 3 blood samples needed for the laboratory in Newcastle, these will still be used for the study and for exploratory analyses. I understand that no more information will be collec after I withdraw unless I give my permission.	
5	I understand that relevant sections of my medical notes and data collected during the study, may be looked by individuals from Newcastle University Clinical Trials Unit, from regulatory authorities or from the NHS Trust, where it is relevant to my taking part in this research. I give permission for these individuals to have access to my records.	
6	I understand that any personal information collected about me for the study will be kept confidential and not be made public. Information from the study will be published in medical journals and at research meetings. I understand that I will not be directly identified in the published results.	
7	I consent for the summary of the results to be sent to me when the study has finished.	
8	I agree for my name, address and contact telephone number and email address (if you have one) to be given to the study team. This is to allow the study team to contact me to inform me of how much anti-thyroid drug I need to take.<br>The email address is ideally required to send communications about the study.	
9	I agree to my General Practitioner being informed about my taking part in this study.	
10	I agree to use the forms of contraception that have been explained to me (if I am sexually active).	
11	I understand that the information collected about me will be used to support other research in the future, and may be shared anonymously with other researchers.	
12	I agree to take part in the above study	
13	I give my permission for a copy of this consent form to be sent securely to the Newcastle Clinical Trials Unit for checking. This is for safety purposes.	

To be answered by Female Participants only

1	I understand that I will need to provide 2 urine samples during the study to make sure that I am not pregnant. I understand that this is for safety purposes.	

______________________ ______________________ ______________

Name of participant **Signature** **Date**

______________________ ______________________ ______________

Name of person taking consent **Signature** **Date**

When completed 1 copy for participant, 1 for researcher site file and 1 (original) to be kept in medical notes and 1 copy to send securely to Newcastle Clinical Trials Unit.
