# Supplementary material for: Adjuvant rituximab, a potential treatment for the young patient with Graves’ hyperthyroidism (RiGD): study protocol for a single-arm, single-stage, phase II trial
Source: BMJ Open. 2019 Jan 21;9(1):e024705. doi: 10.1136/bmjopen-2018-024705 (PMC6347892; doi:10.1136/bmjopen-2018-024705)
Supplement: Author's manuscript [file bmjopen-2018-024705.draft_revisions.pdf]

BMJ Open is committed to open peer review. As part of this commitment we make the peer review history of every article we publish publicly available.

When an article is published we post the peer reviewers' comments and the authors' responses online. We also post the versions of the paper that were used during peer review. These are the versions that the peer review comments apply to.

The versions of the paper that follow are the versions that were submitted during the peer review process. They are not the versions of record or the final published versions. They should not be cited or distributed as the published version of this manuscript.

BMJ Open is an open access journal and the full, final, typeset and author-corrected version of record of the manuscript is available on our site with no access controls, subscription charges or pay-per-view fees (http://bmjopen.bmj.com).

If you have any questions on BMJ Open's open peer review process please email info.bmjopen@bmj.com

BMJ Open

Adjuvant Rituximab, a potential treatment for the young patient with Graves' hyperthyroidism (RiGD): study protocol for a single arm, single stage, phase II trial

Journal:	*BMJ Open*
Manuscript ID	bmjopen-2018-024705
Article Type:	Protocol
Date Submitted by the Author:	09-Jun-2018

Complete List of Authors:	Cole, Michael<br>Hynes, Ann Marie<br>Howel, Denise; Newcastle University<br>Hall, Lesley<br>Abinun, Mario<br>Allahabadia, Amit<br>Barrett, Timothy; Birmingham Childrens HospitalUniversity of Birmingham, Diabetes Unit Institute of Child Health<br>Boelaert, K; University of Birmingham, Institute of Metabolism and Systems Research<br>Drake, Amanda; The University of Edinburgh , Centre for Cardiovascular Science<br>Dimitri, Paul; Sheffield Children's NHS Foundation Trust, Paediatric Endocrinology<br>Kirk, Jeremy<br>Zammitt, Nicola<br>Pearce, Simon; University of Newcastle, School of Clinical Medical Sciences<br>Cheetham, Tim; newcastle University, Paediatric Endocrinology;

Keywords:	Thyroid disease < DIABETES & ENDOCRINOLOGY, IMMUNOLOGY, Paediatric endocrinology < PAEDIATRICS, Clinical trials < THERAPEUTICS

SCHOLARONE™
Manuscripts

Adjuvant Rituximab, a potential treatment for the young patient with Graves' hyperthyroidism (RiGD): study protocol for a single arm, single stage, phase II trial

Michael Cole[1], Ann Marie Hynes[2], Denise Howel[1], Lesley Hall[2], Mario Abinun[3], Amit Allahabadia[4], Timothy Barrett[5], Kristien Boelaert[6], Amanda J Drake[7], Paul Dimitri[8], Jeremy Kirk[9], Nicola Zammitt[10], Simon Pearce[11], Tim Cheetham[12*]

*Correspondence: Tim Cheetham (tim.cheetham@nuth.nhs.uk)

[1] Institute of Health and Society, Newcastle University, The Baddiley-Clark Building, Richardson Road, Newcastle upon Tyne, NE2 4AX, UK.
[2] Newcastle Clinical Trials Unit, Newcastle University, 1-4 Claremont Terrace, Newcastle Upon Tyne, NE2 4AE, UK.
[3] Institute of Cellular Medicine, Newcastle University, Great North Children's Hospital, Newcastle upon Tyne Hospitals NHS Foundation Trust, Newcastle upon Tyne, NE1 4LP, UK.
[4] Academic Directorate of Diabetes and Endocrinology, B230, B Floor, Royal Hallamshire Hospital, Glossop Road, Sheffield, S10 2JF, UK.
[5] c/o Diabetes unit, Birmingham Children's Hospital, Steelhouse Lane, Birmingham, B4 6NH, UK.
[6] Institute of Metabolism and Systems Research, College of Medical and Dental Sciences, Institute of Biomedical Research, Floor 2, University of Birmingham, Birmingham, B15 2TT, UK.
[7] Centre for Cardiovascular Science, Queen's Medical Research Institute, 47 Little France Crescent, Edinburgh, EH16 4TJ, UK.
[8] The Academic Unit of Child Health, Sheffield Children's NHS Trust Western Bank, Sheffield, S10 2TH, UK.
[9] c/o Endocrine Dept, Birmingham Children's Hospital, Steelhouse Lane, Birmingham, B4 6NH, UK.
[10] Edinburgh Centre for Endocrinology & Diabetes, Royal Infirmary of Edinburgh, 51 Little France Crescent, Edinburgh, EH16 4SA, UK.
[11] Institute of Genetic Medicine, Newcastle University, Central Parkway, Newcastle upon Tyne, NE1 3BZ, UK.
[12] Institute of Genetic Medicine, Newcastle University c/o Department of Paediatric Endocrinology, Great North Children's Hospital, Newcastle upon Tyne Hospitals NHS Foundation Trust, Newcastle upon Tyne, NE1 4LP, UK.

For peer review only

For peer review only - http://bmjopen.bmj.com/site/about/guidelines.xhtml

Email addresses of authors:

Michael	Cole	michael.cole@ncl.ac.uk
Ann Marie	Hynes	h.annmarie@gmail.com
Denise	Howel	denise.howel@ncl.ac.uk
Lesley	Hall	lesley.hall@ncl.ac.uk
Mario	Abinun	mario.abinun@ncl.ac.uk
Amit	Allahabadia	amit.allahabadia@sth.nhs.uk
Timothy	Barrett	t.g.barrett@bham.ac.uk
Kristien	Boelaert	k.boelaert@bham.ac.uk
Amanda	Drake	mandy.drake@ed.ac.uk
Paul	Dimitri	paul.dimitri@sch.nhs.uk
Jeremy	Kirk	jeremy.kirk@bch.nhs.uk
Nicola	Zammitt	nicola.zammitt@nhslothian.scot.nhs.uk
Simon	Pearce	s.h.s.pearce@ncl.ac.uk
Tim	Cheetham	tim.cheetham@nuth.nhs.uk

For peer review only

For peer review only - http://bmjopen.bmj.com/site/about/guidelines.xhtml

Abstract

Introduction

Graves' disease is a challenging condition for the young person and their family. The excess thyroid hormone generated by autoimmune stimulation of the TSH receptor on the thyroid gland can have a profound impact on well-being. Managing the young person with Graves' hyperthyroidism is more difficult than in older people because the side-effects of conventional treatment are more significant in this age group and because the disease tends not to resolve spontaneously in the short to medium term. New immunomodulatory agents are now available and anti-B cell monoclonal antibody Rituximab is of particular interest because it targets the cells that manufacture the antibodies that stimulate the thyroid gland in Graves'.

Methods and analysis

The trial aims to establish whether the combination of a single dose of Rituximab (500mg) and a 12 month course of antithyroid drug (usually carbimazole) can result in a meaningful increase in the proportion of patients in remission at 2 years, the primary endpoint. A single-stage Phase II A'Hern design is used. 27 patients aged 12 to 20 years with newly presenting Graves' hyperthyroidism will be recruited. Markers of immune function including lymphocyte numbers and antibody levels (total and specific) will be collected regularly throughout the trial.

Ethics and Dissemination

For peer review only

For peer review only - http://bmjopen.bmj.com/site/about/guidelines.xhtml

The availability of novel immunomodulatory agents that have a reassuring safety record in young people provides an opportunity to explore their efficacy in patients with Graves' hyperthyroidism. This trial will determine whether the immunomodulatory medication, Rituximab, will facilitate remission above and beyond that observed with anti-thyroid drug alone. A meaningful increase in the expected proportion of young patients entering remission when managed according to the trial protocol will justify consideration of a Phase III trial. The results of this trial will be distributed at international endocrine meetings, in the peer-reviewed literature and via patient support groups.

Trial registration: ISRCTN 20381716. Registered on 3 November 2016.

https://doi.org/10.1186/ISRCTN20381716

Keywords

Graves' disease, Rituximab, CD20, Thyroid hormone, Thyroid Receptor antibodies (TRAb) Thyrotropin (TSH), Thyroid Hormone.

For peer review only

For peer review only - http://bmjopen.bmj.com/site/about/guidelines.xhtml

Strengths and limitations

- This is a group of patients in whom current therapy does not usually result in disease resolution. Hence there is a significant unmet need.
- The behaviour of the disease in the young patient with Graves' hyperthyroidism in terms of likelihood of remission following anti-thyroid (thionamide) drug is consistent between reported studies. This will help us to comment on the potential impact of the trial intervention in this exploratory trial without studying a large number of patients.
- We will be looking at a range of markers of immune function which may help to establish some of the factors that predict response to intervention in this group of patients.
- It is possible that there is an immunomodulatory effect of Rituximab that will not be detected because the trial duration of 2 years is too short.
- The likelihood of remission may be different in a 12 year old patient with Graves' hyperthyroidism versus a 20 year old patient. This consideration has not been factored into the trial design.

For peer review only

For peer review only - http://bmjopen.bmj.com/site/about/guidelines.xhtml

Introduction

Graves' hyperthyroidism, an autoimmune disorder, has an annual incidence of 1 in 10,000 adolescents (~700 per year) in the UK [1]. The standard first-line treatment is the anti-thyroid drug (ATD) carbimazole (CBZ) which prevents the thyroid gland from manufacturing thyroid hormone and has an immunomodulatory effect [2]. Approximately 50% of adults will remit following a standard 2 year course of ATD compared to around 25% of children and adolescents. In addition, the side-effects of CBZ are more prevalent in the young with 20% experiencing adverse events that range from relatively minor problems such as rashes through to potentially life-threatening agranulocytosis [3,4]. Establishing a euthyroid state can be difficult in the growing person, made more difficult by poor medication concordance in some young people [5,6]. Avoiding relapse close to key life events such as examinations can result in prolonged courses of ATD therapy. Most young people will ultimately require thyroid gland excision (total thyroidectomy) or thyroid gland ablation with radio-iodine (RI) but these interventions may be associated with additional risks in the young person and do not represent a cure because the patient is then dependent on lifelong levothyroxine replacement [7,8]. Hence, there is a pressing need to develop interventions that can cure a disease that can have major life-long implications [8].

Modern immunomodulatory agents have the potential to ameliorate or 'switch off' the immune response to produce durable remission in patients with Graves' hyperthyroidism. Rituximab (RTX), a chimeric anti-B cell monoclonal antibody (MAb) targeting the surface molecule CD20, leads to reductions in B lymphocyte populations lasting for around 6 months in more than 95% of people following one or two doses [9]. CD20 is only expressed on pre-B lymphocytes and mature B cells; it is not expressed on B lymphocyte stem cells or the committed plasma cell. Circulating B lymphocytes are bound by the antibody and removed

For peer review only - http://bmjopen.bmj.com/site/about/guidelines.xhtml

via antibody-dependent cellular cytotoxicity, complement-mediated lysis and apoptosis. RTX has been successfully used to treat B cell malignancies, with more than 1 million patients treated worldwide in recent years [10]. It has also been used in a range of autoimmune disorders because of its favourable safety profile in adults and children [11,12].

RTX has recognised disease modifying activity both in people with organ-specific and non-organ specific immune-mediated disorders [11-17]. B-cell depletion with RTX has been shown to have disease-modifying effects in rheumatoid arthritis, systemic lupus erythematosus (SLE), autoimmune thrombocytopenia, myasthenia gravis and polyangiitis with granulomatosis including classical T-cell mediated autoimmune diseases such as multiple sclerosis and type 1 diabetes. B cell depletion at sites of active inflammation reduces antigen-presentation to autoreactive T lymphocytes, leading to amelioration of the cytotoxic autoimmune attack. Whilst autoreactive B cells may return in some patients following RTX [18] the impact on disease outcome is not closely linked to circulating antibody levels and the diverse function of B cells including their impact on T cell activity may explain why there may be an effect of Rituximab many years post-administration [19-22].

B lymphocyte depleting immunotherapy appears to have disease-modifying activity in adults with Graves' hyperthyroidism; RTX administration has been associated with encouraging remission rates with approximately 50% of thyrotoxic adults in case reports and case series becoming euthyroid following treatment with RTX [23-28]. Many of these patients were selected on the basis of more severe disease with relapsed Graves' hyperthyroidism or aggressive orbitopathy. The management of cases where there was no clear response to RTX were characterised by early intervention with ATD, surgery or RI within the first year after RTX therapy which may have obscured a beneficial effect of immunotherapy on thyroid status. Graves' hyperthyroidism and Graves' orbitopathy (GO) have several

For peer review only - http://bmjopen.bmj.com/site/about/guidelines.xhtml

immunopathogenic features in common, including shared autoantigens and several studies indicate that RTX has the potential to modify the natural history of thyroid eye disease [29-31]. However, this has not been a consistent observation with one randomised trial showing no impact of RTX on GO [32]. The difference in trial outcome has been reviewed [33] and the differences could reflect factors such as the younger age of those recruited to the trial in Italy [30].

RTX has an excellent safety record in the young and has been used extensively in paediatric practice to treat autoimmune cytopaenias, juvenile dermatomyositis, juvenile idiopathic arthritis, systemic lupus erythematodes (SLE) and renal disorders with significant side-effects uncommon [16,34]. Rates of infection appear to be low [14]. Hypogammaglobulinemia is a possible but very rare side-effect in young people and may reflect an underlying but previously unrecognised immunodeficiency [35]. The rare complication, progressive multi-focal leukoencephalopathy, has been seen almost exclusively in adult patients with complex disorders who have received multiple immune suppressants (eg. those with B cell malignancies, multiorgan refractory SLE).

The aim of this clinical trial is to explore whether RTX administration will have a beneficial impact on the disease course in young people presenting with Graves' hyperthyroidism. Specifically, we have set out to establish whether a single 500mg dose of RTX, when administered together with a 12 month course of ATD, will increase remission rates in young people with Graves' hyperthyroidism.

Previous treatment regimens in adults with Graves' disease or GO have typically used between 1000 and 2000mg RTX administered in divided doses. In contrast, a single dose of 500mg RTX was used in a recent trial of patients with GO [30]. The investigators in this trial

For peer review only - http://bmjopen.bmj.com/site/about/guidelines.xhtml

subsequently reduced the dose of RTX to 100mg in some of their trial patients because complete peripheral B cell depletion was still seen with this smaller dose. Our selection of 500mg of RTX was a pragmatic decision bearing in mind the established efficacy in many disorders of 2 x 1000mg doses versus the observation that a profound effect on B cell numbers was still observed with a much smaller dose. We were also keen to reduce the complexity associated with repeated visits to hospital for an intravenous infusion. By using a modest dose of 500mg RTX on one occasion we anticipated a meaningful effect on immune system function but a reduction in potential side-effects [27].

A course of ATD will be also prescribed in addition to a single dose of RTX so that patients are rendered clinically and biochemically euthyroid whilst the immune modulating effects of RTX alter the underlying immune disease process. A dose-titration ATD regimen will be used to reduce the likelihood of a subject having to stop ATD medication whilst still hyperthyroid [36]. ATD will be stopped before 12 months has elapsed if patients become hypothyroid whilst on the smallest dose of ATD (5mg CBZ alternate days). As well as preventing thyroid hormone production, ATD has a specific immunomodulatory effect including reducing thyroid autoantibody concentrations during treatment [37,38]. These effects may involve alteration of thyroid antigen structure [39], inhibition of pro-inflammatory cytokines or inhibition of T lymphocytes by other potential mechanisms [40]. An additive or indeed synergistic effect of RTX and ATD is theoretically possible via combined effects on autoantibody generation and the combination of RTX and ATD may increase remission rates above those observed in studies to date. Another advantage of the immunomodulatory approach to autoimmune hyperthyroidism is the potential for intervention to reduce the likelihood of longer-term hypothyroidism [41,42].

For peer review only - http://bmjopen.bmj.com/site/about/guidelines.xhtml

Methods and Analysis

Trial objectives

The primary objective is to establish whether a single 500mg dose of RTX, when administered together with a 12 month course of ATD, is likely to result in a meaningful improvement in the proportion of young people with Graves' hyperthyroidism entering disease remission.

The secondary objectives are to examine: i) the relationship between thyrotropin receptor antibody (TRAb) titre and thyroid hormone status (thyroid stimulating hormone [TSH], free thyroxine [FT4] and free tri-iodothyronine [FT3]) in recruited subjects at the beginning and end of the trial; 2) whether immune cellular response is related to disease outcome by examining the relationship between time to recovery of B cell numbers measured in peripheral blood to within the local normal reference range and thyroid hormone status at the end of the trial; 3) whether total dose of ATD is related to disease outcome; 4) the time taken for TSH concentrations to normalise post RTX and to describe thyroid status, as assessed by TSH, FT4 and FT3 concentrations (normal or abnormal), in the period between cessation of ATD therapy and the end of the trial in each patient; 5) the safety of the trial treatment regimen by determining the nature and frequency of adverse events.

Trial Design

This is an investigator-initiated, open-label, single arm, single stage, phase II trial using an A'Hern design [43] to determine whether the proposed new treatment is likely to meet a minimum level of efficacy before comparing it to standard treatment in a randomised trial. A single arm design has been chosen because use of a control arm would require a much larger

For peer review only - http://bmjopen.bmj.com/site/about/guidelines.xhtml

sample size resulting in a longer, more expensive trial when the potential benefit of RTX is, as yet, unknown. The design requires specification of the smallest remission rate, which if true, would clearly imply that the treatment warrants further investigation; and the largest remission rate below which we would not wish to proceed to a larger definitive trial.

Trial Setting

This is a multi-centre trial based in seven paediatric and seven adult tertiary endocrine units in the cities of Birmingham, Edinburgh, Newcastle, Leeds, Sheffield, Cardiff and Southampton UK and one secondary paediatric unit in Doncaster, UK.

For peer review only

Inclusion Criteria

- Excess thyroid hormone concentrations at diagnosis: elevated free FT3 and / or FT4 (based on local assay).
- Suppressed (un-recordable) TSH (based on local assay).
- Patients between the ages of 12-20 years inclusive who are less than 6 weeks from the initiation of ATD treatment (carbimazole or propylthiouracil) for the first time.
- Elevated thyroid binding inhibitory immunoglobulin or thyroid receptor antibodies (TRAb including thyroid binding inhibiting immunoglobulin [TBII]) based on local assay. Patients may or may not have a raised Thyroid Peroxidase (TPO) antibody titre.
- All patients must be willing to use effective forms of contraception for 12 months post-treatment with RTX.
- If females are of childbearing potential, they must have a negative pregnancy test at screening. This will need to be repeated on the day of RTX administration if more than 7 days has elapsed since the screening visit or a negative pregnancy test.
- Able and willing to adhere to a 2 year trial period.

For peer review only - http://bmjopen.bmj.com/site/about/guidelines.xhtml

Exclusion criteria

- Previous episodes of autoimmune thyroid disease
- Patients with an active, severe infection (e.g. tuberculosis, sepsis and opportunistic infections) or severely immunocompromised patients.
- Patients with known allergy or contraindication to carbimazole and propylthiouracil.
- Participants with previous use of immunosuppressive or cytotoxic drugs (including RTX and methylprednisolone but excluding inhaled glucocorticoid and oral glucocorticoid for asthma or topical glucocorticoid for eczema).
- Chromosomal disorders known to be associated with an increased risk of autoimmune thyroid disease including Downs' syndrome and Turners' syndrome.
- Pregnancy, planned pregnancy during the trial period or current breast-feeding
- Absence of informed consent from parent/legal guardian for participants age <16 years.
- Participants with significant chronic cardiac, respiratory or renal disorder or non-autoimmune liver disease.
- Participants with known allergy or contraindication to RTX or methylprednisolone.
- Participants with evidence of Hepatitis B/C infection, assessed by determining hepatitis 'B' surface antigen (HBsAg) status, hepatitis 'B' Core antibody (HB Core antibody) status and hepatitis 'C' virus antibody (HCV antibody) status.
- Participants in families who know they will be moving out of the catchment areas during the 2 years following RTX treatment.
- Participants currently involved in any other clinical trial of an investigational medicinal product (IMP) or who have taken an IMP within 30 days prior to trial entry.

Patient identification

For peer review only - http://bmjopen.bmj.com/site/about/guidelines.xhtml

Potentially eligible patients will be referred by their local clinician (GP, paediatrician, physician or endocrinologist) or through self-referral via national organisations such as the British Thyroid Foundation (BTF) to a clinician with delegated responsibility to discuss the trial.

Screening, recruitment and consent

Informed consent discussions will be undertaken by appropriate site staff, with an opportunity for the patient and the parent/guardian to ask questions. Following receipt of the PIS, participants will be given a minimum of 24 hours to decide whether or not they would like to participate. As part of the consent discussions the requirement to use effective contraception must be discussed. The PIS states that after the patient has given consent, they will be required to provide a blood sample and undergo a pregnancy test (if female) to confirm that they are not pregnant and that there is no evidence of Hepatitis B/C infection. Some sites will also need to carry out human immunodeficiency virus (HIV) screening and Quantiferon tests to confirm that the patients do not have Tuberculosis and HIV (as per their local clinical policies) before a patient can be confirmed as eligible. Those wishing to take part will provide written informed consent/assent prior to trial specific procedures/investigations.

For peer review only

If thyroid antibody status (TRAb or TBII) was not determined at diagnosis then this will be assessed at this consent and screening visit. The patient will be classified as a screen fail if they are found not to be eligible at this point. Ineligible patients will be notified via telephone and not asked to come in for a further trial visit. A screening log will be kept securely at each of the recruiting units (paediatric and adult units) to document details of eligible patients who have screen failed or declined to participate in the trial, including any reasons available. The right to refuse to participate without giving reasons will be respected.

For peer review only - http://bmjopen.bmj.com/site/about/guidelines.xhtml

Intervention

RTX will be administered as a 500mg dose by slow intravenous infusion under cover with methylprednisolone 125mg IV, chlorpheniramine 10mg IV and 500mg (12 to 16 years of age) or 1g (>16 years) of paracetamol (PO). The RTX infusion will be started at a rate of 50mg/hr increasing by 50mg/hr every half hour if tolerated; otherwise the infusion will either be maintained at the current rate or stopped and then restarted after 30 minutes. If the RTX infusion is tolerated and the rate increased as above then the infusion will take 3.25 hours. Symptoms which are considered intolerable are angioedema, low blood pressure and difficulty breathing; if any are observed the treatment will be stopped immediately, only to be restarted once symptoms have resolved. Vital signs, including blood pressure will be measured every 30 minutes during the RTX infusion and for 2 hours afterwards.

RTX administered may only take place following confirmation that the patient is hepatitis B/C antibody negative and within 7 days of a negative test for pregnancy (females). Administration of live vaccines is contraindicated or not recommended for at least 6 months following RTX administration.

For peer review only - http://bmjopen.bmj.com/site/about/guidelines.xhtml

Table 1. CBZ dose adjustment according to changing thyroid hormone status

Week / stage of trial	Thyroid hormone level: FT4 and FT3 (pmol/l)	CBZ dose (mg)
Presentation		20mg CBZ once daily (consider 10mg in the case of mild hyperthyroidism (FT3<10).
Week 4	Biochemically hypothyroid (low FT4, FT3 not raised)	Reduce to 5mg
	FT3 normal and FT4 falling	Consider reducing from 20mg to 10mg or 10 to 5mg
	FT3 elevated	Continue prior dose
Week 8	Biochemically hypothyroid (low FT4, FT3 not raised)	Reduce to 20mg, 10mg, 5mg or reduce CBZ to 5mg alternate days
	FT3 normal and FT4 falling	Reduce from 20mg to 10mg, 10mg to 5mg or remain on 5mg
	FT3 elevated but improving	Continue prior dose
	FT3 raised or increasing further	Consider increasing CBZ by 10mg daily
Week 12 and beyond	Biochemically hypothyroid (low FT4, FT3 not raised)	Reduce to 10mg, 5mg or reduce CBZ to 5mg alternate days
	FT3 normal (or elevated with low FT4)	Continue current dose
	FT3 elevated	Consider increasing CBZ by 10mg daily or 5mg daily if previously euthyroid
Week 16 and beyond	TSH elevation (> 4mU/l) (TSH takes precedence over thyroid hormone levels)	Reduce CBZ dose by 5 to 10mg daily
	TSH still suppressed	Follow guide above for Week 12

For peer review only

For peer review only - http://bmjopen.bmj.com/site/about/guidelines.xhtml

ATD therapy (Carbimazole and Propylthiouracil)

A 12 month course of ATD will be administered to induce a euthyroid state in the months following diagnosis. Therapy will normally be with CBZ which can be administered once daily. PTU is second line therapy because of the increased likelihood of hepatic failure with this drug. It is proposed that CBZ dose be titrated against prevailing thyroid function tests in order to maintain a euthyroid state. Clinicians will be encouraged to follow the schedule outlined in Table 1 when administering ATD to patients enrolled into this clinical trial although the managing clinician can elect not to follow the above framework if their assessment of the overall clinical picture suggests that this is in the patients' best interests. PTU can be administered on the basis that 5mg of CBZ is equivalent to 50mg PTU. If patients become hypothyroid (with an elevated TSH and low FT4/FT3 level) on the smallest daily dose of CBZ (5mg) or PTU (50mg) then the ATD can be administered on alternate days. If patients are still hypothyroid then the ATD will be stopped before 12 months has elapsed following RTX therapy.

Any participants who develop unacceptable toxicity to CBZ (notably neutropenia with a neutrophil count less than 0.5 $x10^9$/l) will stop ATD immediately. Participants with a neutrophil count between 0.5 and 1.0 can remain on ATD if well. The neutrophil count should be repeated after approximately 1 to 2 weeks or as clinically indicated to confirm that it has not fallen to less than 0.5 $x10^9$/l. PTU can be commenced as a replacement ATD in the case of adverse events that do not involve hepatic dysfunction or neutropenia. The increased risk of liver dysfunction on PTU will be discussed with participants and their families prior to commencing PTU. Patients on PTU will have liver function checked at each visit and PTU stopped if ALT or bilirubin are elevated (2x above the upper limit of normal). If patients are unable to tolerate ATD then it may be feasible to monitor patients off treatment or manage

For peer review only - http://bmjopen.bmj.com/site/about/guidelines.xhtml

them symptomatically with beta blockade on the basis that RTX may alter the disease natural history and result in remission without further ATD. This option can be discussed with the participant / the participant and their family. Other potential means of maintaining a euthyroid state can be discussed with the trial management team.

For peer review only

For peer review only - http://bmjopen.bmj.com/site/about/guidelines.xhtml

Table 2: Schedule of events

Assessments/intervention	Trial period														
	Screening / consent	Trial intervention	Follow-up												
	Week -6 to 0	0	4	8	12	16	20	28	36	44	52	65	78	91	104
Confirmation of eligibility	X	X													
Informed consent and assent	X														
HBsAG, HB Core & HCV antibodies	X														
Pregnancy test (females)	X	X									X				
Medical history		X													
Clinical examination		X	X	X	X	X	X	X	X	X	X	X	X	X	X
Height		X						X			X		X		X
Weight		X	X	X	X	X	X	X	X	X	X	X	X	X	X
RTX infusion		X													
ATD regimen		X	X	X	X	X	X	X	X	X	X				
ATD compliance			X	X	X	X	X	X	X	X	X				
Concomitant medication		X	X	X	X	X	X	X	X	X	X	X	X	X	X
Adverse and Serious Adverse Event		X	X	X	X	X	X	X	X	X	X	X	X	X	X
Thyroid function (TSH, FT4 & FT3)		X	X	X	X	X	X	X	X	X	X	X	X	X	X
Liver function (ALT and bilirubin) if on PTU		X	X	X	X	X	X	X	X	X	X	X	X	X	X
Thyroid antibodies (TRAB & TPO)		X			X			X	X		X				X
Lymphocyte subsets*		X	X		X			X	X		X				X
Full blood count		X	X		X			X	X		X				X
Immunoglobulin levels (IgG, IgM, IgA)		X			X			X	X		X				X
Specific antibody levels (tetanus, Hib, pneumococcus)		X									X				X
Thyroid Function (TSH, FT4 & FT3) – central analysis		X									X				X
Serum for exploratory analyses		X									X				X

*Lymphocyte subsets: T cells (CD3), Helper T cells (CD4), Cytotoxic T cells (CD8), B cells (CD19) and class switch B cells (CD27+ve IgD-)
ATD dose is adjusted at each visit if required.

For peer review only

For peer review only - http://bmjopen.bmj.com/site/about/guidelines.xhtml

Outcome measures

Primary

The number of subjects in remission at 2 years following a single dose of RTX and a 12 month course of ATD is the primary trial endpoint: this is equivalent to the number who have not relapsed. Subjects will be deemed to have relapsed if they are receiving any concomitant ATD medication in the second year post RTX administration, or have undergone surgical (thyroidectomy) or RI treatment because of hyperthyroidism at any time following RTX administration. They will also be deemed to have relapsed if serum TSH is less than the lower limit of the normal laboratory reference range and serum FT3 is above the upper limit of the normal laboratory reference range at 2 years.

Secondary

1. TRAb titre and related thyroid hormone status at the time of RTX administration and 2 years post RTX.
2. Time to recovery of B cell lymphocyte numbers in peripheral blood to the normal local lab reference range in relation to thyroid hormone status.
3. Cumulative dose of ATD (mg/kg) in relation to thyroid hormone status 2 years post RTX.
4. Time taken for TSH and thyroid hormone concentrations to normalise to within the local laboratory reference range post RTX and describe biochemical thyroid status in the period between cessation of ATD and 2 years post RTX in each patient.
5. The frequency and nature of adverse events.

For peer review only - http://bmjopen.bmj.com/site/about/guidelines.xhtml

Visit details and Assessments

Following screening and consent, RTX is administered at the baseline visit (week 0). Subsequent visits are scheduled until 24 months, details are provided in table 2. In addition, after each visit in the first 12 months, participants are contacted by telephone to document changes in ATD dosages. Calls should be completed within 10 days of the visit.

HBsAG, HB Core antibody and HCV antibody status is checked at screening/consent to establish hepatitis status to determine if the patient be entered into the trial. A full blood count is conducted to look for evidence of change in white cell number (a recognised side-effect of ATD). Lymphocyte subsets including T cells (CD3+), helper T cells (CD4+), cytotoxic T cells (CD8+), B cells (CD19+) and class switch B cells (CD27+ IgD-), and serum immunoglobulin (Ig) levels (IgG, IgM, IgA) are measured to look at the impact of RTX (anti-B cell MAb) on B and T cell populations and immunoglobulin levels (produced by plasma cells that differentiate from B cells).

Specific vaccine antibodies (against tetanus, haemophilus influenzae type B (Hib), and pneumococcus) are assessed at baseline and at 12 and 24 months, to look for evidence of falling levels as a consequence of the intervention.

At each visit, concomitant medications are recorded, an adverse event check is performed and thyroid function (TSH, FT4 & FT3) is assessed.

We plan to undertake exploratory analyses of immune system function on blood samples obtained prior to the RTX infusion at baseline and then after 12 months and 24 months. This will include measurement of a range of novel factors involved in immune system activity including B lymphocyte activating factor (BAFF), a proliferating-inducing ligand (APRIL)

For peer review only - http://bmjopen.bmj.com/site/about/guidelines.xhtml

and B lymphocyte chemoattractant (CXCL13). This data will be analysed separately from the main trial.

Withdrawal

Participants have the right to withdraw from the trial at any time, including during the single infusion of RTX, without having to give a reason. The patient will still be asked to complete follow up. Sites will need to record the amount of RTX that was administered prior to the patients' withdrawal. Investigator sites should try to ascertain the reason for withdrawal and document this reason within the Case Report Form and participant's medical notes.

As this is a single dose administration it is not anticipated that the investigator would withdraw a patient from treatment (the infusion would be slowed down accordingly –see ***Intervention*** section). If the RTX infusion has to be stopped completely and cannot be recommenced then those subjects receiving less than 100mg RTX will be reviewed according to the protocol for safety reasons only. Should a participant withdraw from the trial then every effort will be made to obtain follow-up data, with the permission of the patient.

Participants who withdraw from the trial will not be replaced.

Sample Size

Formal justification to proceed to a larger randomised trial is based upon observing a minimum number achieving remission 2 years post RTX administration; this number is referred to as the critical number [43]. The critical number depends upon the desired and unacceptable remission rates, set at 40% and 20%, respectively, and the error levels set at 10% alpha (type I) and 20% beta (type II). The remission rate corresponding to the critical number will lie between 40% and 20% and will be specified and stored separately in the

For peer review only - http://bmjopen.bmj.com/site/about/guidelines.xhtml

Statistics TMF. If the true remission rate is 40% there is an 80% chance (power) of proceeding to a further trial; if the true remission rate is 20%, there is a small 10% (alpha) chance of proceeding to a further trial. With these parameters the target recruitment is 27 patients who will receive RTX. This is the smallest number of patients which satisfies the design criteria, assuming a 10% loss to follow-up.

Statistical Analysis

Patients will receive a single dose of RTX once they have fulfilled the inclusion and exclusion criteria. In the event that RTX is not well tolerated, only patients who were able to receive more than 100mg of RTX will be included in the statistical analysis.

Analysis of the Primary Outcome Measure

The number of patients in remission 2 years after RTX administration will be compared to the critical number. This is equivalent to a formal comparison of the hypotheses that the remission rate is greater than or equal to 40% as opposed to less than or equal to 20%. The primary outcome measure, remission rate, will be presented with a one sided 90% lower bound. If this phase II trial provides evidence that the true remission rate is plausibly 40% or more, 2 years after a single dose of RTX and a 12 month course of ATD, then this will indicate a likely effect of RTX on disease outcome and justify a randomised efficacy evaluation of this adjuvant RTX regimen.

Analysis of Secondary Outcome Measures

1. The distribution of TRAb titres will be compared between patients in and out of remission by graphical summary, numerical summary statistics (median and interquartile range) and a Mann-Whitney test.

For peer review only - http://bmjopen.bmj.com/site/about/guidelines.xhtml

2. Time to recovery of peripheral blood B cell lymphocyte numbers (CD 19+ cells) to above 70% of the patient's pre RTX level in relation to thyroid hormone status. This will be compared between patients in and out of remission by graphical summary, numerical summary statistics (median and interquartile range) and a Mann-Whitney test.
3. Cumulative dose of ATD (Carbimazole) in mg/kg in relation to thyroid hormone status. This will be compared between patients in and out of remission by graphical summary, numerical summary statistics (median and interquartile range) and a Mann-Whitney test.
4. We will assess the time taken for TSH concentrations to normalise (increase above 0.1mU/l) post RTX and describe biochemical thyroid status, as assessed by TSH, FT4 and FT3 concentration, in the period between cessation of ATD therapy at the end of year one and the end of the trial. Specifically we will plot the time taken for TSH to normalise in individual patients and determine the proportion of time that TSH, FT4 and FT3 concentrations are within the normal reference range in the period between ATD stopping (end of year 1) and trial end (end of year 2).
5. The frequency and nature of adverse events.

Criteria for the Premature Termination of the Trial

The criteria for stopping the trial will be if a patient dies from a RTX treatment related infection, or if 2 patients experience the same SUSAR which has severe or life-threatening consequences.

Safety reporting

We do not aim to collect adverse events such as minor trauma (scratches, cuts) in patients who are otherwise well. Adverse events will otherwise be classified according to whether they are:

For peer review only - http://bmjopen.bmj.com/site/about/guidelines.xhtml

1. Infections (minor = self-limiting infection such as the common cold, modest= non-self-limiting where an antibiotic is prescribed but the clinical course and recovery is not related to the underlying condition and its' treatment or severe where antibiotic therapy +/- other therapy is required with the infection related to drug effects on immune function).
2. Immune function related such as neutropaenia.
3. Related to the skin e.g. rashes.
4. Related to the musculo-skeletal system such as joint pain.
5. Related to gastro-intestinal upset.
6. Related to hepatic function.
7. Others

The trial will be stopped if a patient dies from a RTX treatment related infection or if 2 patients experience the same SUSAR which has severe or life-threatening consequences.

According to section 4.6 of the SmPC (Mabthera [the product name for RTX] 100mg and 500mg Concentrate for Solution for Infusion), RTX should not be administered to pregnant women. The SmPC states that "Rituximab should not be administered to pregnant women unless the possible benefit outweighs the potential risk". Due to the long retention time of RTX in B-cell depleted patients, women of childbearing potential need to use effective contraceptive methods during treatment and for 12 months following RTX therapy (in line with the SmPC for RTX). If a female participant becomes pregnant within 12 months of taking the RTX drug the details of the pregnancy needs to be reported to the Chief Investigator, Trial Manager and Sponsor within 24 hours of the site learning of its occurrence on the pregnancy reporting form.

For peer review only - http://bmjopen.bmj.com/site/about/guidelines.xhtml

If a patient or their partner (where the participant is male) becomes pregnant, within 12 months of taking RTX, the pregnancy must be reported as per the trial specific guidance document for pregnancy reporting and followed up to completion of pregnancy.

Ethics and Dissemination

The trial has received a favourable ethical opinion (North East - Tyne and Wear South Research Ethics committee, reference 16/NE/0253, EudraCT number 2016-000209-35).Trial oversight consists of Trial Steering Committee (TSC), Data Monitoring Committee (DMC) and Trial Management Group (TMG). The TSC comprises an Independent Chair and three other independent members including a statistician. The DMC consists of three independent members, an endocrinologist (Chair), immunologist and a statistician. The TMG will be responsible for overseeing the progress of the trial. The day-to-day management of the trial will be co-ordinated by Newcastle Clinical Trials Unit.

The results of this trial will be distributed widely at international endocrine meetings including the European Society for Paediatric Endocrinology annual meeting. The results will be published the peer-reviewed literature and distributed via patient support groups including the British Thyroid Foundation.

For peer review only

For peer review only - http://bmjopen.bmj.com/site/about/guidelines.xhtml

Discussion

Graves' disease remains a challenge for young people and their families with significant disadvantages associated with all standard treatment modalities. Most young people will ultimately be treated with thyroid hormone replacement which is not ideal from a practical, financial and quality of life perspective [44]. The management of Graves' hyperthyroidism is currently under review by NICE.

This is a proof of concept trial that may be the precursor to a phase III trial. The limitations of the trial design include the need to adopt a largely pragmatic approach to the selection of the Rituximab dose and the fact that an impact on disease course may not be detected by a 2 year trial. On the other hand, patients who remit by 2 years may still be at risk of relapse at a later stage. There is also a risk that patients will experience side-effects from ATD therapy that mean this has to be stopped. If so, then definitive treatment may be required before the impact of RTX on the disease course can be elucidated. There is also a need to be cautious about assuming that remission rates following a course of ATD in a 12 year old patient with Graves' hyperthyroidism are similar to those in someone who is 20 years of age. Remission rate may therefore reflect the age of trial participants as well as the impact of the treatment regimen.

We feel that there are three disadvantages from the patient's perspective when taking part in this trial. Firstly, the infusion involves a day spent in hospital. The second disadvantage is the potential risk of adverse events, notably infections, although the literature does not suggest significant issues in an otherwise healthy and immune competent group of subjects like young patients with Graves' hyperthyroidism who have received one or two doses of Rituximab. Finally there is the uncertainty about whether the intervention will impact on disease outcome favourably.

For peer review only - http://bmjopen.bmj.com/site/about/guidelines.xhtml

The number of clinic visits will be similar to patients with Graves' managed routinely and will help to ensure that patients biochemistry is monitored carefully.

ATD does not cure young Graves' patients in the short term and is only associated with resolution of the hyperthyroid state in a minority of patients after several years of treatment. Surgery and radioiodine remove or destroy the gland and hence are not a cure either. Rituximab provides the prospect of an earlier remission in patients with Graves' hyperthyroidism who are therefore less likely to experience the side-effects of ATD. Rituximab may cure patients who would otherwise not have been cured by ATD and it may reduce the likelihood of long-term hypothyroidism. Re-purposing rituximab for this indication would mean an immediately translatable therapy with a well-established and favourable side-effect profile, and a likely reduction in costs now that it is off-patent.

For peer review only

For peer review only - http://bmjopen.bmj.com/site/about/guidelines.xhtml

Trial status

This manuscript is based upon trial protocol version 4.0 dated 27th November 2017. The RiGD trial opened to recruitment in October 2016 and is due to close to recruitment in October 2018.

For peer review only

For peer review only - http://bmjopen.bmj.com/site/about/guidelines.xhtml

List of abbreviations

Abbreviation	Definition
AE	Adverse Event
ALT	Alanine transaminase
AR	Adverse Reaction
ATD	Antithyroid Drug
BTF	British Thyroid Foundation
CA	Competent Authority
CBZ	Carbimazole
CI	Chief Investigator
CRF	Case Report Form
CTA	Clinical Trial Authorisation
CTIMP	Clinical Trial of an Investigational Medicinal Product
DGH	District General Hospital
DLT	Dose Limiting Toxicity
DMC	Data Monitoring Committee
DSUR	Development Safety Update Report
eCRF	Electronic case report form
EudraCT	European Clinical Trials Database
EMA	European Medicines Agency
FT3	Free Tri-iodothyronine
FT4	Free Thyroxine
GCP	Good Clinical Practice
GD	Graves' Disease
GO	Graves' Orbitopathy
HBsAg	Hepatitis 'B' surface antigen
HB Core	Hepatitis 'B' Core
HCV	Hepatitis 'C' virus
HIV	Human Immunodeficiency Virus
HRA	Health Research Authority
HTA	Human Tissue Authority
HTAct	Human Tissue Act
IB	Investigator Brochure
ICF	Informed Consent Form
ICH	International Conference on Harmonisation
IMP	Investigational Medicinal Product
ISF	Investigator Site File
ISRCTN	International Standard Randomised Controlled Trials Number
MA	Marketing Authorisation
MHRA	Medicines and Healthcare products Regulatory Agency
MP	Methylprednisolone
NCTU	Newcastle Clinical Trials Unit
NHS	National Health Service
PI	Principal Investigator
PIS	Participant Information Sheet
PK	Pharmacokinetic
PTU	Propylthiouracil

For peer review only - http://bmjopen.bmj.com/site/about/guidelines.xhtml
For peer review only

QA	Quality Assurance
QC	Quality Control
QP	Qualified Person
R&D	Research & Development
REC	Research Ethics Committee
RSI	Reference Safety Information
RI	Radio-iodine
RTX	Rituximab (Mabthera ®)
SAE	Serious Adverse Event
SAR	Serious Adverse Reaction
SDV	Source Data Verification
SLE	Systemic Lupus Erythematosus
SOP	Standard Operating Procedure
SmPC	Summary of Product Characteristics
SSI	Site Specific Information
SUSAR	Suspected Unexpected Serious Adverse Reaction
TBII	Thyroid Binding Inhibiting Immunoglobulin
TMF	Trial Master File
TMG	Trial Management Group
TPO	Thyroid Peroxidase
TRAb	Thyroid receptor antibody
TSC	Trial Steering Committee
TSH	Thyroid Stimulating Hormone
TSHR	Thyroid Stimulating Hormone Receptor

For peer review only

For peer review only - http://bmjopen.bmj.com/site/about/guidelines.xhtml

Declarations

Ethics approval and consent to participate

REC approval was the 15th of September 2016. The first patient was consented on the 4th November 2016.

Consent for publication

Not applicable

Availability of data and materials

Not applicable

Competing interests

The authors declare that they have no competing interests

Funding

The trial is funded by a grant from the Medical Research Council Biomedical Catalyst: Developmental Pathway Funding Scheme (DPFS).

Authors' contributions

SP and TC had the original idea for the trial. The trial protocol was developed by TC with contributions from SP, MC, DH, MA, AMH, LH and AD. The manuscript was primarily drafted by MC, DH, MA and TC, but all authors were in involved in revising the manuscript, and gave final approval of the submitted version.

Acknowledgements

We would like to acknowledge the guidance provided by the local young person's advisory group and by the British Thyroid Foundation when the trial documentation was being

For peer review only - http://bmjopen.bmj.com/site/about/guidelines.xhtml

For peer review only

developed. We would also like to acknowledge the ongoing support provided by the clinical and research teams from the endocrine units in Cardiff, Doncaster, Leeds and Southampton who opened as participating sites after the trial commenced.

References

1 Flynn RWV, MacDonald TM, Morris AD, Jung RT, Leese GP. The thyroid epidemiology, audit, and research study: Thyroid dysfunction in the general population. J Clin Endocr Metab. 2004;89:3879-3884.

2 McDonald DO, Pearce SHS. Thyroid peroxidase forms thionamide-sensitive homodimers: relevance for immunomodulation of thyroid autoimmunity. J Mol Med-Jmm. 2009;87:971-980.

3 Rivkees SA, Stephenson K, Dinauer C. Adverse Events Associated with Methimazole Therapy of Graves' Disease in Children. International Journal of Pediatric Endocrinology. 2010;2010:176970.

4 Rivkees SA, Sklar C, Freemark M. The Management of Graves' Disease in Children, with Special Emphasis on Radioiodine Treatment. The Journal of Clinical Endocrinology & Metabolism. 1998;83:3767-3776.

5 Lazar L, Kalter-Leibovici O, Pertzelan A, Weintrob N, Josefsberg Z, Phillip M. Thyrotoxicosis in Prepubertal Children Compared with Pubertal and Postpubertal Patients. The Journal of Clinical Endocrinology & Metabolism. 2000;85:3678-3682.

6 Glaser NS, Styne DM, Organization of Pediatric Endocrinologists of Northern California Collaborative Graves' Disease Study G. Predicting the likelihood of remission in children with Graves' disease: a prospective, multicenter study. Pediatrics. 2008;121:e481-488.

7 Cheetham T, Bliss R. Treatment options in the young patient with Graves' disease. Clinical endocrinology. 2016;85:161-164.

8 Franklyn JA, Boelaert K. Thyrotoxicosis. The Lancet. 2012;379:1155-1166.

For peer review only - http://bmjopen.bmj.com/site/about/guidelines.xhtml

9 Leandro MJ, Cambridge G, Ehrenstein MR, Edwards JC. Reconstitution of peripheral blood B cells after depletion with rituximab in patients with rheumatoid arthritis. Arthritis Rheum. 2006;54.

10 Boross P, Leusen JHW. Mechanisms of action of CD20 antibodies. Am J Cancer Res. 2012;2:676-690.

11 Edwards JCW, Szczepański L, Szechiński J, Filipowicz-Sosnowska A, Emery P, Close DR, Stevens RM, Shaw T. Efficacy of B-Cell–Targeted Therapy with Rituximab in Patients with Rheumatoid Arthritis. New England Journal of Medicine. 2004;350:2572-2581.

12 Jansson AF, Sengler C, Kuemmerle-Deschner J, Gruhn B, Kranz AB, Lehmann H, Kleinert D, Pape L, Girschick HJ, Foeldvari I, *et al.* B cell depletion for autoimmune diseases in paediatric patients. Clinical Rheumatology. 2011;30:87-97.

13 Alexeeva EI, Valieva SI, Bzarova TM, Semikina EL, Isaeva KB, Lisitsyn AO, Denisova RV, Chistyakova EG. Efficacy and safety of repeat courses of rituximab treatment in patients with severe refractory juvenile idiopathic arthritis. Clin Rheumatol. 2011;30:1163-1172.

14 Kavcic M, Fisher BT, Seif AE, Li Y, Huang YS, Walker D, Aplenc R. Leveraging administrative data to monitor rituximab use in 2875 patients at 42 freestanding children's hospitals across the United States. The Journal of pediatrics. 2013;162:1252-1258, 1258.e1251.

15 Lippe BM, Landaw EM, Kaplan SA. Hyperthyroidism in children treated with long term medical therapy: twenty-five percent remission every two years. The Journal of clinical endocrinology and metabolism. 1987;64:1241-1245.

16 Rheumatology BSfPaA. Clinical Guidelines. 2017 [cited 7 November 2017]. Available from: http://80.87.12.43/bspar/guidelines/default.aspx

17 Hauser SL, Waubant E, Arnold DL, Vollmer T, Antel J, Fox RJ, Bar-Or A, Panzara M, Sarkar N, Agarwal S, *et al.* B-cell depletion with Rituximab in relapsing-remitting multiple sclerosis. New England Journal of Medicine. 2008;358:676-688.

18 Chamberlain N, Massad C, Oe T, Cantaert T, Herold KC, Meffre E, Pathway TDT. Rituximab does not reset defective early B cell tolerance checkpoints. Journal of Clinical Investigation. 2016;126:282-287.

For peer review only - http://bmjopen.bmj.com/site/about/guidelines.xhtml

19 Kamei K, Ishikura K, Sako M, Aya K, Tanaka R, Nozu K, Kaito H, Nakanishi K, Ohtomo Y, Miura K*, et al.* Long-term outcome of childhood-onset complicated nephrotic syndrome after a multicenter, double-blind, randomized, placebo-controlled trial of rituximab. Pediatr Nephrol. 2017;32:2071-2078.

20 Oved JH, Lee CSY, Bussel JB. Treatment of Children with Persistent and Chronic Idiopathic Thrombocytopenic Purpura: 4 Infusions of Rituximab and Three 4-Day Cycles of Dexamethasone. J Pediatr-Us. 2017;191:225-231.

21 Tak PP, Rigby W, Rubbert-Roth A, Peterfy C, van Vollenhoven RF, Stohl W, Healy E, Hessey E, Reynard M, Shaw T. Sustained inhibition of progressive joint damage with rituximab plus methotrexate in early active rheumatoid arthritis: 2-year results from the randomised controlled trial IMAGE. Annals of the Rheumatic Diseases. 2012;71:351-357.

22 Verstappen GM, Kroese FGM, Meiners PM, Corneth OB, Huitema MG, Haacke EA, van der Vegt B, Arends S, Vissink A, Bootsma H*, et al.* B Cell Depletion Therapy Normalizes Circulating Follicular Th Cells in Primary Sjogren Syndrome. J Rheumatol. 2017;44.

23 Ohye H, Minagawa A, Noh JY, Mukasa K, Kunii Y, Watanabe N, Matsumoto M, Suzuki M, Yoshihara A, Ito K*, et al.* Antithyroid drug treatment for graves' disease in children: a long-term retrospective study at a single institution. Thyroid : official journal of the American Thyroid Association. 2014;24:200-207.

24 El Fassi D, Nielsen CH, Bonnema SJ, Hasselbalch HC, Hegedus L. B lymphocyte depletion with the monoclonal antibody rituximab in Graves' disease: a controlled pilot study. The Journal of clinical endocrinology and metabolism. 2007;92:1769-1772.

25 Salvi M, Vannucchi G, Campi I, Rossi S, Bonara P, Sbrozzi F, Guastella C, Avignone S, Pirola G, Ratiglia R*, et al.* Efficacy of rituximab treatment for thyroid-associated ophthalmopathy as a result of intraorbital B-cell depletion in one patient unresponsive to steroid immunosuppression. European journal of endocrinology. 2006;154:511-517.

26 El Fassi D, Nielsen CH, Hasselbalch HC, Hegedus L. Treatment-resistant severe, active Graves' ophthalmopathy successfully treated with B lymphocyte depletion. Thyroid : official journal of the American Thyroid Association. 2006;16:709-710.

27 Salvi M, Vannucchi G, Campi I, Curro N, Dazzi D, Simonetta S, Bonara P, Rossi S, Sina C, Guastella C*, et al.* Treatment of Graves' disease and associated ophthalmopathy with the anti-CD20 monoclonal antibody rituximab: an open study. European journal of endocrinology. 2007;156:33-40.

For peer review only - http://bmjopen.bmj.com/site/about/guidelines.xhtml

28 Heemstra KA, Toes RE, Sepers J, Pereira AM, Corssmit EP, Huizinga TW, Romijn JA, Smit JW. Rituximab in relapsing Graves' disease, a phase II study. European journal of endocrinology. 2008;159:609-615.

29 Mitchell AL, Gan EH, Morris M, Johnson K, Neoh C, Dickinson AJ, Perros P, Pearce SH. The effect of B cell depletion therapy on anti-TSH receptor antibodies and clinical outcome in glucocorticoid-refractory Graves' orbitopathy. Clinical endocrinology. 2013;79:437-442.

30 Salvi M, Vannucchi G, Curro N, Campi I, Covelli D, Dazzi D, Simonetta S, Guastella C, Pignataro L, Avignone S, *et al.* Efficacy of B-cell targeted therapy with rituximab in patients with active moderate to severe Graves' orbitopathy: a randomized controlled study. The Journal of clinical endocrinology and metabolism. 2015;100:422-431.

31 Wang CJ, Ning QY, Jin K, Xie JJ, Ye J. Does rituximab improve clinical outcomes of patients with thyroid-associated ophthalmopathy? A systematic review and meta-analysis. Bmc Ophthalmol. 2018;18.

32 Stan MN, Garrity JA, Leon BGC, Prabin T, Bradley EA, Bahn RS. Randomized Controlled Trial of Rituximab in Patients With Graves' Orbitopathy. J Clin Endocr Metab. 2015;100:432-441.

33 Stan MN, Salvi M. MANAGEMENT OF ENDOCRINE DISEASE Rituximab therapy for Graves' orbitopathy - lessons from randomized control trials. European journal of endocrinology. 2017;176:R101-R109.

34 Iijima K, Sako M, Nozu K, Mori R, Tuchida N, Kamei K, Miura K, Aya K, Nakanishi K, Ohtomo Y, *et al.* Rituximab for childhood-onset, complicated, frequently relapsing nephrotic syndrome or steroid-dependent nephrotic syndrome: a multicentre, double-blind, randomised, placebo-controlled trial. Lancet. 2014;384:1273-1281.

35 Kaplan B, Kopyltsova Y, Khokhar A, Lam F, Bonagura V. Rituximab and immune deficiency: case series and review of the literature. The journal of allergy and clinical immunology In practice. 2014;2:594-600.

36 Abraham P, Avenell A, McGeoch SC, Clark LF, Bevan JS. Antithyroid drug regimen for treating Graves' hyperthyroidism. The Cochrane database of systematic reviews. 2010:Cd003420.

37 McGregor AM, Petersen MM, McLachlan SM, Rooke P, Smith BR, Hall R. Carbimazole and the autoimmune response in Graves' disease. N Engl J Med. 1980;303:302-307.

For peer review only - http://bmjopen.bmj.com/site/about/guidelines.xhtml

For peer review only

38 Marcocci C, Chiovato L, Mariotti S, Pinchera A. Changes of circulating thyroid autoantibody levels during and after the therapy with methimazole in patients with Graves' disease. Journal of endocrinological investigation. 1982;5:13-19.

39 McDonald DO, Pearce SH. Thyroid peroxidase forms thionamide-sensitive homodimers: relevance for immunomodulation of thyroid autoimmunity. Journal of molecular medicine (Berlin, Germany). 2009;87:971-980.

40 Humar M, Dohrmann H, Stein P, Andriopoulos N, Goebel U, Roesslein M, Schmidt R, Schwer CI, Loop T, Geiger KK, *et al.* Thionamides inhibit the transcription factor nuclear factor-kappaB by suppression of Rac1 and inhibitor of kappaB kinase alpha. The Journal of pharmacology and experimental therapeutics. 2008;324:1037-1044.

41 Hamburger JI. Management of hyperthyroidism in children and adolescents. The Journal of clinical endocrinology and metabolism. 1985;60:1019-1024.

42 Wood LC, Ingbar SH. Hypothyroidism as a Late Sequela in Patients with Graves' Disease Treated with Antithyroid Agents. Journal of Clinical Investigation. 1979;64:1429-1436.

43 A'Hern RP. Sample size tables for exact single-stage phase II designs. Statistics in medicine. 2001;20:859-866.

44 Saravanan P, Chau WF, Roberts N, Vedhara K, Greenwood R, Dayan CM. Psychological well-being in patients on 'adequate' doses of L-thyroxine: results of a large, controlled community-based questionnaire study. Clinical endocrinology. 2002;57:577-585.

For peer review only

For peer review only - http://bmjopen.bmj.com/site/about/guidelines.xhtml

BMJ Open

Adjuvant Rituximab, a potential treatment for the young patient with Graves' hyperthyroidism (RiGD): study protocol for a single arm, single stage, phase II trial

Journal:	*BMJ Open*
Manuscript ID	bmjopen-2018-024705.R1
Article Type:	Protocol
Date Submitted by the Author:	25-Sep-2018

Complete List of Authors:	Cole, Michael<br>Hynes, Ann Marie<br>Howel, Denise; Newcastle University<br>Hall, Lesley<br>Abinun, Mario<br>Allahabadia, Amit<br>Barrett, Timothy; Birmingham Childrens HospitalUniversity of Birmingham, Diabetes Unit Institute of Child Health<br>Boelaert, K; University of Birmingham, Institute of Metabolism and Systems Research<br>Drake, Amanda; The University of Edinburgh , Centre for Cardiovascular Science<br>Dimitri, Paul; Sheffield Children's NHS Foundation Trust, Paediatric Endocrinology<br>Kirk, Jeremy<br>Zammitt, Nicola<br>Pearce, Simon; University of Newcastle, School of Clinical Medical Sciences<br>Cheetham, Tim; newcastle University, Paediatric Endocrinology;

**Primary Subject Heading**:	Diabetes and endocrinology
Secondary Subject Heading:	Immunology (including allergy), Medical management, Paediatrics
Keywords:	Thyroid disease < DIABETES & ENDOCRINOLOGY, IMMUNOLOGY, Paediatric endocrinology < PAEDIATRICS, Clinical trials < THERAPEUTICS

SCHOLARONE™
Manuscripts

Adjuvant Rituximab, a potential treatment for the young patient with Graves' hyperthyroidism (RiGD): study protocol for a single arm, single stage, phase II trial

Michael Cole[1], Ann Marie Hynes[2], Denise Howel[1], Lesley Hall[2], Mario Abinun[3], Amit Allahabadia[4], Timothy Barrett[5], Kristien Boelaert[6], Amanda J Drake[7], Paul Dimitri[8], Jeremy Kirk[9], Nicola Zammitt[10], Simon Pearce[11], Tim Cheetham[12]*

*Correspondence: Tim Cheetham (tim.cheetham@nuth.nhs.uk)

[1] Institute of Health and Society, Newcastle University, The Baddiley-Clark Building, Richardson Road, Newcastle upon Tyne, NE2 4AX, UK.
[2] Newcastle Clinical Trials Unit, Newcastle University, 1-4 Claremont Terrace, Newcastle Upon Tyne, NE2 4AE, UK.
[3] Institute of Cellular Medicine, Newcastle University, Great North Children's Hospital, Newcastle upon Tyne Hospitals NHS Foundation Trust, Newcastle upon Tyne, NE1 4LP, UK.
[4] Academic Directorate of Diabetes and Endocrinology, B230, B Floor, Royal Hallamshire Hospital, Glossop Road, Sheffield, S10 2JF, UK.
[5] c/o Diabetes unit, Birmingham Children's Hospital, Steelhouse Lane, Birmingham, B4 6NH, UK.
[6] Institute of Metabolism and Systems Research, College of Medical and Dental Sciences, Institute of Biomedical Research, Floor 2, University of Birmingham, Birmingham, B15 2TT, UK.
[7] Centre for Cardiovascular Science, Queen's Medical Research Institute, 47 Little France Crescent, Edinburgh, EH16 4TJ, UK.
[8] The Academic Unit of Child Health, Sheffield Children's NHS Trust Western Bank, Sheffield, S10 2TH, UK.
[9] c/o Endocrine Dept, Birmingham Children's Hospital, Steelhouse Lane, Birmingham, B4 6NH, UK.
[10] Edinburgh Centre for Endocrinology & Diabetes, Royal Infirmary of Edinburgh, 51 Little France Crescent, Edinburgh, EH16 4SA, UK.
[11] Institute of Genetic Medicine, Newcastle University, Central Parkway, Newcastle upon Tyne, NE1 3BZ, UK.
[12] Institute of Genetic Medicine, Newcastle University c/o Department of Paediatric Endocrinology, Great North Children's Hospital, Newcastle upon Tyne Hospitals NHS Foundation Trust, Newcastle upon Tyne, NE1 4LP, UK.

For peer review only

For peer review only - http://bmjopen.bmj.com/site/about/guidelines.xhtml

Email addresses of authors:

Michael	Cole	michael.cole@ncl.ac.uk
Ann Marie	Hynes	h.annmarie@gmail.com
Denise	Howel	denise.howel@ncl.ac.uk
Lesley	Hall	lesley.hall@ncl.ac.uk
Mario	Abinun	mario.abinun@ncl.ac.uk
Amit	Allahabadia	amit.allahabadia@sth.nhs.uk
Timothy	Barrett	t.g.barrett@bham.ac.uk
Kristien	Boelaert	k.boelaert@bham.ac.uk
Amanda	Drake	mandy.drake@ed.ac.uk
Paul	Dimitri	paul.dimitri@sch.nhs.uk
Jeremy	Kirk	jeremy.kirk@bch.nhs.uk
Nicola	Zammitt	nicola.zammitt@nhslothian.scot.nhs.uk
Simon	Pearce	s.h.s.pearce@ncl.ac.uk
Tim	Cheetham	tim.cheetham@nuth.nhs.uk

For peer review only

For peer review only - http://bmjopen.bmj.com/site/about/guidelines.xhtml

Abstract

Introduction

Graves' disease (Graves' hyperthyroidism) is a challenging condition for the young person and their family. The excess thyroid hormone generated by autoimmune stimulation of the TSH receptor on the thyroid gland can have a profound impact on well-being. Managing the young person with Graves' hyperthyroidism is more difficult than in older people because the side-effects of conventional treatment are more significant in this age group and because the disease tends not to resolve spontaneously in the short to medium term. New immunomodulatory agents are available and the anti-B cell monoclonal antibody Rituximab is of particular interest because it targets cells that manufacture the antibodies that stimulate the thyroid gland in Graves'.

Methods and analysis

The trial aims to establish whether the combination of a single dose of Rituximab (500mg) and a 12 month course of antithyroid drug (usually carbimazole) can result in a meaningful increase in the proportion of patients in remission at 2 years, the primary endpoint. A single-stage Phase II A'Hern design is used. 27 patients aged 12 to 20 years with newly presenting Graves' hyperthyroidism will be recruited. Markers of immune function including lymphocyte numbers and antibody levels (total and specific) will be collected regularly throughout the trial.

Ethics and Dissemination

For peer review only - http://bmjopen.bmj.com/site/about/guidelines.xhtml

The trial will determine whether the immunomodulatory medication, Rituximab, will facilitate remission above and beyond that observed with anti-thyroid drug alone. A meaningful increase in the expected proportion of young patients entering remission when managed according to the trial protocol will justify consideration of a Phase III trial. The trial has received a favourable ethical opinion (North East - Tyne and Wear South Research Ethics committee, reference 16/NE/0253, EudraCT number 2016-000209-35). The results of this trial will be distributed at international endocrine meetings, in the peer-reviewed literature and via patient support groups.

Trial registration: ISRCTN 20381716. Registered on 3 November 2016. https://doi.org/10.1186/ISRCTN20381716

Keywords

Graves' disease, Rituximab, CD20, Thyroid hormone, Thyroid Receptor antibodies (TRAb) Thyrotropin (TSH), Thyroid Hormone.

Trial sponsor: The Newcastle Upon Tyne Hospitals NHS Foundation Trust

For peer review only - http://bmjopen.bmj.com/site/about/guidelines.xhtml

Strengths and limitations

- This is a group of patients in whom current therapy does not usually result in disease resolution with only 20 to 30% remitting after a 2 year course of antithyroid drug treatment with carbimazole. Hence there is a significant unmet need.
- The behaviour of the disease in the young patient with Graves' hyperthyroidism in terms of likelihood of remission following anti-thyroid (thionamide) drug is consistent between reported studies. This will help us to comment on the potential impact of the trial intervention in this exploratory trial without studying a large number of patients.
- We will be looking at a range of markers of immune function which may help to establish some of the factors that predict response to intervention in this group of patients.
- It is possible that there is an immunomodulatory effect of Rituximab that will not be detected because the trial duration of 2 years is too short.
- The likelihood of remission may be different in a 12 year old patient with Graves' hyperthyroidism versus a 20 year old patient. This consideration has not been factored into the trial design.

For peer review only

For peer review only - http://bmjopen.bmj.com/site/about/guidelines.xhtml

Introduction

Graves' hyperthyroidism, an autoimmune disorder, has an annual incidence of 1 in 10,000 adolescents (~700 per year) in the UK [1]. The standard first-line treatment is the anti-thyroid drug (ATD) carbimazole (CBZ) which prevents the thyroid gland from manufacturing thyroid hormone and has an immunomodulatory effect [2]. Whilst CBZ will render most patients biochemically euthyroid in appropriate doses only 50% of adults will remit following a standard 2 year course of ATD. The proportion of children and adolescents entering remission is considerably smaller at around 25% and yet the side-effects of CBZ are more prevalent in the young with 20% experiencing adverse events that range from relatively minor problems such as rashes through to potentially life-threatening agranulocytosis [3,4]. Establishing a euthyroid state can be difficult in the growing person, made more difficult by poor medication concordance in some young people [5,6]. Avoiding relapse close to key life events such as examinations can result in prolonged courses of ATD therapy. Most young people will ultimately require thyroid gland excision (total thyroidectomy) or thyroid gland ablation with radio-iodine (RI) but these interventions may be associated with additional risks in the young person and do not represent a cure because the patient is then dependent on lifelong levothyroxine replacement [7,8]. Hence, there is a pressing need to develop interventions that can cure a disease that can have major life-long implications [8].

Modern immunomodulatory agents have the potential to ameliorate or 'switch off' the immune response to produce durable remission in patients with Graves' hyperthyroidism. Rituximab (RTX), a chimeric anti-B cell monoclonal antibody (MAb) targeting the surface molecule CD20, leads to reductions in B lymphocyte populations lasting for around 6 months in more than 95% of people following one or two doses [9]. CD20 is only expressed on pre-B lymphocytes and mature B cells; it is not expressed on B lymphocyte stem cells or the

For peer review only - http://bmjopen.bmj.com/site/about/guidelines.xhtml

committed plasma cell. Circulating B lymphocytes are bound by the antibody and removed via antibody-dependent cellular cytotoxicity, complement-mediated lysis and apoptosis. RTX has been successfully used to treat B cell malignancies, with more than 1 million patients treated worldwide in recent years [10]. It has also been used in a range of autoimmune disorders because of its favourable safety profile in adults and children [11,12].

RTX has recognised disease modifying activity both in people with organ-specific and non-organ specific immune-mediated disorders [11-17]. B-cell depletion with RTX has been shown to have disease-modifying effects in rheumatoid arthritis, systemic lupus erythematosus (SLE), autoimmune thrombocytopenia, myasthenia gravis and polyangiitis with granulomatosis including classical T-cell mediated autoimmune diseases such as multiple sclerosis and type 1 diabetes. B cell depletion at sites of active inflammation reduces antigen-presentation to autoreactive T lymphocytes, leading to amelioration of the cytotoxic autoimmune attack. Whilst autoreactive B cells may return in some patients following RTX [18] the impact on disease outcome is not closely linked to circulating antibody levels and the diverse function of B cells including their impact on T cell activity may explain why there may be an effect of Rituximab many years post-administration [19-22].

B lymphocyte depleting immunotherapy appears to have disease-modifying activity in adults with Graves' hyperthyroidism; RTX administration has been associated with encouraging remission rates with approximately 50% of thyrotoxic adults in case reports and case series becoming euthyroid following treatment with RTX [23-28]. Many of these patients were selected on the basis of more severe disease with relapsed Graves' hyperthyroidism or aggressive orbitopathy. The management of cases where there was no clear response to RTX were characterised by early intervention with ATD, surgery or RI within the first year after RTX therapy which may have obscured a beneficial effect of immunotherapy on thyroid

For peer review only - http://bmjopen.bmj.com/site/about/guidelines.xhtml

status. Graves' hyperthyroidism and Graves' orbitopathy (GO) have several immunopathogenic features in common, including shared autoantigens and several studies indicate that RTX has the potential to modify the natural history of thyroid eye disease [29-31]. However, this has not been a consistent observation with one randomised trial showing no impact of RTX on GO [32]. The difference in trial outcome has been reviewed [33] and the differences could reflect factors such as the younger age of those recruited to the trial in Italy [30].

RTX has an excellent safety record in the young and has been used extensively in paediatric practice to treat autoimmune cytopaenias, juvenile dermatomyositis, juvenile idiopathic arthritis, systemic lupus erythematodes (SLE) and renal disorders with significant side-effects uncommon [16,34]. Rates of infection appear to be low [14]. Hypogammaglobulinemia is a possible but very rare side-effect in young people and may reflect an underlying but previously unrecognised immunodeficiency [35]. The rare complication, progressive multi-focal leukoencephalopathy, has been seen almost exclusively in adult patients with complex disorders who have received multiple immune suppressants (eg. those with B cell malignancies, multiorgan refractory SLE).

The aim of this clinical trial is to explore whether RTX administration will have a beneficial impact on the disease course in young people presenting with Graves' hyperthyroidism. Specifically, we have set out to establish whether a single 500mg dose of RTX, when administered together with a 12 month course of ATD, will increase remission rates in young people with Graves' hyperthyroidism.

Previous treatment regimens in adults with Graves' disease or GO have typically used between 1000 and 2000mg RTX administered in divided doses. In contrast, a single dose of

For peer review only - http://bmjopen.bmj.com/site/about/guidelines.xhtml

For peer review only

500mg RTX was used in a recent trial of patients with GO [30]. The investigators in this trial subsequently reduced the dose of RTX to 100mg in some of their trial patients because complete peripheral B cell depletion was still seen with this smaller dose. Our selection of 500mg of RTX was a pragmatic decision bearing in mind the established efficacy in many disorders of 2 x 1000mg doses versus the observation that a profound effect on B cell numbers was still observed with a much smaller dose. We were also keen to reduce the complexity associated with repeated visits to hospital for an intravenous infusion. By using a modest dose of 500mg RTX on one occasion we anticipated a meaningful effect on immune system function but a reduction in potential side-effects [27].

A course of ATD will be also prescribed in addition to a single dose of RTX so that patients are rendered clinically and biochemically euthyroid whilst the immune modulating effects of RTX alter the underlying immune disease process. A dose-titration ATD regimen will be used to reduce the likelihood of a subject having to stop ATD medication whilst still hyperthyroid [36]. ATD will be stopped before 12 months has elapsed if patients become hypothyroid whilst on the smallest dose of ATD (5mg CBZ alternate days). As well as preventing thyroid hormone production, ATD has a specific immunomodulatory effect including reducing thyroid autoantibody concentrations during treatment [37,38]. These effects may involve alteration of thyroid antigen structure [39], inhibition of pro-inflammatory cytokines or inhibition of T lymphocytes by other potential mechanisms [40]. An additive or indeed synergistic effect of RTX and ATD is theoretically possible via combined effects on autoantibody generation and the combination of RTX and ATD may increase remission rates above those observed in studies to date. Another advantage of the immunomodulatory approach to autoimmune hyperthyroidism is the potential for intervention to reduce the likelihood of longer-term hypothyroidism [41,42].

For peer review only - http://bmjopen.bmj.com/site/about/guidelines.xhtml

Methods and Analysis

Trial objectives

The primary objective is to establish whether a single 500mg dose of RTX, when administered together with a 12 month course of ATD, is likely to result in a meaningful improvement in the proportion of young people with Graves' hyperthyroidism entering disease remission.

The secondary objectives are to examine: i) the relationship between thyrotropin receptor antibody (TRAb) titre and thyroid hormone status (thyroid stimulating hormone [TSH], free thyroxine [FT4] and free tri-iodothyronine [FT3]) in recruited subjects at the beginning and end of the trial; 2) whether immune cellular response is related to disease outcome by examining the relationship between time to recovery of B cell numbers measured in peripheral blood to within the local normal reference range and thyroid hormone status at the end of the trial; 3) whether total dose of ATD is related to disease outcome; 4) the time taken for TSH concentrations to normalise post RTX and to describe thyroid status, as assessed by TSH, FT4 and FT3 concentrations (normal or abnormal), in the period between cessation of ATD therapy and the end of the trial in each patient; 5) the safety of the trial treatment regimen by determining the nature and frequency of adverse events.

Trial Design

This is an investigator-initiated, open-label, single arm, single stage, phase II trial using an A'Hern design [43] to determine whether the proposed new treatment is likely to meet a minimum level of efficacy before comparing it to standard treatment in a randomised trial. A

For peer review only - http://bmjopen.bmj.com/site/about/guidelines.xhtml

single arm design has been chosen because use of a control arm would require a much larger sample size resulting in a longer, more expensive trial when the potential benefit of RTX is, as yet, unknown. The design requires specification of the smallest remission rate, which if true, would clearly imply that the treatment warrants further investigation; and the largest remission rate below which we would not wish to proceed to a larger definitive trial.

Trial Setting

This is a multi-centre trial based in seven paediatric and seven adult tertiary endocrine units in the cities of Birmingham, Edinburgh, Newcastle, Leeds, Sheffield, Cardiff and Southampton UK and one secondary paediatric unit in Doncaster, UK.

Inclusion Criteria

- Excess thyroid hormone concentrations at diagnosis: elevated free FT3 and / or FT4 (based on local assay).
- Suppressed (un-recordable) TSH (based on local assay).
- Patients between the ages of 12-20 years inclusive who are less than 6 weeks from the initiation of ATD treatment (carbimazole or propylthiouracil) for the first time.
- Elevated thyroid binding inhibitory immunoglobulin or thyroid receptor antibodies (TRAb including thyroid binding inhibiting immunoglobulin [TBII]) based on local assay. Patients may or may not have a raised Thyroid Peroxidase (TPO) antibody titre.
- All patients must be willing to use effective forms of contraception for 12 months post-treatment with RTX.
- If females are of childbearing potential, they must have a negative pregnancy test at screening. This will need to be repeated on the day of RTX administration if more than 7 days has elapsed since the screening visit or a negative pregnancy test.

For peer review only - http://bmjopen.bmj.com/site/about/guidelines.xhtml

- Able and willing to adhere to a 2 year trial period.

Exclusion criteria

- Previous episodes of autoimmune thyroid disease
- Patients with an active, severe infection (e.g. tuberculosis, sepsis and opportunistic infections) or severely immunocompromised patients.
- Patients with known allergy or contraindication to carbimazole and propylthiouracil.
- Participants with previous use of immunosuppressive or cytotoxic drugs (including RTX and methylprednisolone but excluding inhaled glucocorticoid and oral glucocorticoid for asthma or topical glucocorticoid for eczema).
- Chromosomal disorders known to be associated with an increased risk of autoimmune thyroid disease including Downs' syndrome and Turners' syndrome.
- Pregnancy, planned pregnancy during the trial period or current breast-feeding
- Absence of informed consent from parent/legal guardian for participants age <16 years.
- Participants with significant chronic cardiac, respiratory or renal disorder or non-autoimmune liver disease.
- Participants with known allergy or contraindication to RTX or methylprednisolone.
- Participants with evidence of Hepatitis B/C infection, assessed by determining hepatitis 'B' surface antigen (HBsAg) status, hepatitis 'B' Core antibody (HB Core antibody) status and hepatitis 'C' virus antibody (HCV antibody) status.
- Participants in families who know they will be moving out of the catchment areas during the 2 years following RTX treatment.
- Participants currently involved in any other clinical trial of an investigational medicinal product (IMP) or who have taken an IMP within 30 days prior to trial entry.

For peer review only - http://bmjopen.bmj.com/site/about/guidelines.xhtml

Patient identification

Potentially eligible patients will be referred by their local clinician (GP, paediatrician, physician or endocrinologist) or through self-referral via national organisations such as the British Thyroid Foundation (BTF) to a clinician with delegated responsibility to discuss the trial.

Screening, recruitment and consent

Informed consent discussions will be undertaken by appropriate site staff, with an opportunity for the patient and the parent/guardian to ask questions. Following receipt of the PIS, participants will be given a minimum of 24 hours to decide whether or not they would like to participate. As part of the consent discussions the requirement to use effective contraception must be discussed. The PIS states that after the patient has given consent, they will be required to provide a blood sample and undergo a pregnancy test (if female) to confirm that they are not pregnant and that there is no evidence of Hepatitis B/C infection. Some sites will also need to carry out human immunodeficiency virus (HIV) screening and Quantiferon tests to confirm that the patients do not have Tuberculosis and HIV (as per their local clinical policies) before a patient can be confirmed as eligible. Those wishing to take part will provide written informed consent/assent prior to trial specific procedures/investigations. The information sheets and consent / assent forms are shown in Appendices 1a, 1b, 1c and 1d.

If thyroid antibody status (TRAb or TBII) was not determined at diagnosis then this will be assessed at this consent and screening visit. The patient will be classified as a screen fail if they are found not to be eligible at this point. Ineligible patients will be notified via telephone and not asked to come in for a further trial visit. A screening log will be kept securely at each of the recruiting units (paediatric and adult units) to document details of eligible patients who

For peer review only - http://bmjopen.bmj.com/site/about/guidelines.xhtml

have screen failed or declined to participate in the trial, including any reasons available. The right to refuse to participate without giving reasons will be respected.

Intervention

RTX will be administered as a 500mg dose by slow intravenous infusion under cover with methylprednisolone 125mg IV, chlorpheniramine 10mg IV and 500mg (12 to 16 years of age) or 1g (>16 years) of paracetamol (PO). The RTX infusion will be started at a rate of 50mg/hr increasing by 50mg/hr every half hour if tolerated; otherwise the infusion will either be maintained at the current rate or stopped and then restarted after 30 minutes. If the RTX infusion is tolerated and the rate increased as above then the infusion will take 3.25 hours. Symptoms which are considered intolerable are angioedema, low blood pressure and difficulty breathing; if any are observed the treatment will be stopped immediately, only to be restarted once symptoms have resolved. Vital signs, including blood pressure will be measured every 30 minutes during the RTX infusion and for 2 hours afterwards.

RTX administered may only take place following confirmation that the patient is hepatitis B/C antibody negative and within 7 days of a negative test for pregnancy (females). Administration of live vaccines is contraindicated or not recommended for at least 6 months following RTX administration.

For peer review only - http://bmjopen.bmj.com/site/about/guidelines.xhtml

Table 1. CBZ dose adjustment according to changing thyroid hormone status

Week / stage of trial	Thyroid hormone level: FT4 and FT3 (pmol/l)	CBZ dose (mg)
Presentation		20mg CBZ once daily (consider 10mg in the case of mild hyperthyroidism (FT3<10).
Week 4	Biochemically hypothyroid (low FT4, FT3 not raised)	Reduce to 5mg
	FT3 normal and FT4 falling	Consider reducing from 20mg to 10mg or 10 to 5mg
	FT3 elevated	Continue prior dose
Week 8	Biochemically hypothyroid (low FT4, FT3 not raised)	Reduce to 20mg, 10mg, 5mg or reduce CBZ to 5mg alternate days
	FT3 normal and FT4 falling	Reduce from 20mg to 10mg, 10mg to 5mg or remain on 5mg
	FT3 elevated but improving	Continue prior dose
	FT3 raised or increasing further	Consider increasing CBZ by 10mg daily
Week 12 and beyond	Biochemically hypothyroid (low FT4, FT3 not raised)	Reduce to 10mg, 5mg or reduce CBZ to 5mg alternate days
	FT3 normal (or elevated with low FT4)	Continue current dose
	FT3 elevated	Consider increasing CBZ by 10mg daily or 5mg daily if previously euthyroid
Week 16 and beyond	TSH elevation (> 4mU/l) (TSH takes precedence over thyroid hormone levels)	Reduce CBZ dose by 5 to 10mg daily
	TSH still suppressed	Follow guide above for Week 12

For peer review only - http://bmjopen.bmj.com/site/about/guidelines.xhtml

ATD therapy (Carbimazole and Propylthiouracil)

A 12 month course of ATD will be administered to induce a euthyroid state in the months following diagnosis. Therapy will normally be with CBZ which can be administered once daily. PTU is second line therapy because of the increased likelihood of hepatic failure with this drug. It is proposed that CBZ dose be titrated against prevailing thyroid function tests in order to maintain a euthyroid state. Clinicians will be encouraged to follow the schedule outlined in Table 1 when administering ATD to patients enrolled into this clinical trial although the managing clinician can elect not to follow the above framework if their assessment of the overall clinical picture suggests that this is in the patients' best interests. PTU can be administered on the basis that 5mg of CBZ is equivalent to 50mg PTU. If patients become hypothyroid (with an elevated TSH and low FT4/FT3 level) on the smallest daily dose of CBZ (5mg) or PTU (50mg) then the ATD can be administered on alternate days. If patients are still hypothyroid then the ATD will be stopped before 12 months has elapsed following RTX therapy.

Any participants who develop unacceptable toxicity to CBZ (notably neutropenia with a neutrophil count less than 0.5 $x10^9/l$) will stop ATD immediately. Participants with a neutrophil count between 0.5 and 1.0 can remain on ATD if well. The neutrophil count should be repeated after approximately 1 to 2 weeks or as clinically indicated to confirm that it has not fallen to less than 0.5 $x10^9/l$. PTU can be commenced as a replacement ATD in the case of adverse events that do not involve hepatic dysfunction or neutropenia. The increased risk of liver dysfunction on PTU will be discussed with participants and their families prior to commencing PTU. Patients on PTU will have liver function checked at each visit and PTU stopped if ALT or bilirubin are elevated (2x above the upper limit of normal). If patients are unable to tolerate ATD then it may be feasible to monitor patients off treatment or manage

For peer review only - http://bmjopen.bmj.com/site/about/guidelines.xhtml

them symptomatically with beta blockade on the basis that RTX may alter the disease natural history and result in remission without further ATD. This option can be discussed with the participant / the participant and their family. Other potential means of maintaining a euthyroid state can be discussed with the trial management team.

Patients who relapse in the second year of the trial will be encouraged to return to antithyroid drug in the first instance.

For peer review only

For peer review only - http://bmjopen.bmj.com/site/about/guidelines.xhtml

Table 2: Schedule of events

Assessments/intervention	Trial period														
	Screening / consent	Trial intervention	Follow-up												
	Week -6 to 0	0	4	8	12	16	20	28	36	44	52	65	78	91	104
Confirmation of eligibility	X	X													
Informed consent and assent	X														
HBsAG, HB Core & HCV antibodies	X														
Pregnancy test (females)	X	X									X				
Medical history		X													
Clinical examination		X	X	X	X	X	X	X	X	X	X	X	X	X	X
Height		X						X			X		X		X
Weight		X	X	X	X	X	X	X	X	X	X	X	X	X	X
RTX infusion		X													
ATD regimen		X	X	X	X	X	X	X	X	X	X				
ATD compliance			X	X	X	X	X	X	X	X	X				
Concomitant medication		X	X	X	X	X	X	X	X	X	X	X	X	X	X
Adverse and Serious Adverse Event		X	X	X	X	X	X	X	X	X	X	X	X	X	X
Thyroid function (TSH, FT4 & FT3)		X	X	X	X	X	X	X	X	X	X	X	X	X	X
Liver function (ALT and bilirubin) if on PTU		X	X	X	X	X	X	X	X	X	X	X	X	X	X
Thyroid antibodies (TRAB & TPO)		X			X			X	X		X				X
Lymphocyte subsets*		X	X		X			X	X		X				X
Full blood count		X	X		X			X	X		X				X
Immunoglobulin levels (IgG, IgM, IgA)		X			X			X	X		X				X
Specific antibody levels (tetanus, Hib, pneumococcus)		X									X				X
Thyroid Function (TSH, FT4 & FT3) – central analysis		X									X				X
Serum for exploratory analyses		X									X				X

*Lymphocyte subsets: T cells (CD3), Helper T cells (CD4), Cytotoxic T cells (CD8), B cells (CD19) and class switch B cells (CD27+ve IgD-)
ATD dose is adjusted at each visit if required.

For peer review only - http://bmjopen.bmj.com/site/about/guidelines.xhtml

Outcome measures

Primary

The number of subjects in remission at 2 years following a single dose of RTX and a 12 month course of ATD is the primary trial endpoint: this is equivalent to the number who have not relapsed. Subjects will be deemed to have relapsed if they are receiving any concomitant ATD medication in the second year post RTX administration, or have undergone surgical (thyroidectomy) or RI treatment because of hyperthyroidism at any time following RTX administration. They will also be deemed to have relapsed if serum TSH is less than the lower limit of the normal laboratory reference range and serum FT3 is above the upper limit of the normal laboratory reference range at 2 years.

Secondary

1. TRAb titre and related thyroid hormone status at the time of RTX administration and 2 years post RTX.
2. Time to recovery of B cell lymphocyte numbers in peripheral blood to the normal local lab reference range in relation to thyroid hormone status.
3. Cumulative dose of ATD (mg/kg) in relation to thyroid hormone status 2 years post RTX.
4. Time taken for TSH and thyroid hormone concentrations to normalise to within the local laboratory reference range post RTX and describe biochemical thyroid status in the period between cessation of ATD and 2 years post RTX in each patient.
5. The frequency and nature of adverse events.

For peer review only - http://bmjopen.bmj.com/site/about/guidelines.xhtml

Visit details and Assessments

Following screening and consent, RTX is administered at the baseline visit (week 0). Subsequent visits are scheduled until 24 months, details are provided in table 2. In addition, after each visit in the first 12 months, participants are contacted by telephone to document changes in ATD dosages. Calls should be completed within 10 days of the visit.

HBsAG, HB Core antibody and HCV antibody status is checked at screening/consent to establish hepatitis status to determine if the patient be entered into the trial. A full blood count is conducted to look for evidence of change in white cell number (a recognised side-effect of ATD). Lymphocyte subsets including T cells (CD3+), helper T cells (CD4+), cytotoxic T cells (CD8+), B cells (CD19+) and class switch B cells (CD27+ IgD-), and serum immunoglobulin (Ig) levels (IgG, IgM, IgA) are measured to look at the impact of RTX (anti-B cell MAb) on B and T cell populations and immunoglobulin levels (produced by plasma cells that differentiate from B cells).

Specific vaccine antibodies (against tetanus, haemophilus influenzae type B (Hib), and pneumococcus) are assessed at baseline and at 12 and 24 months, to look for evidence of falling levels as a consequence of the intervention.

At each visit, concomitant medications are recorded, an adverse event check is performed and thyroid function (TSH, FT4 & FT3) is assessed.

We plan to undertake exploratory analyses of immune system function on blood samples obtained prior to the RTX infusion at baseline and then after 12 months and 24 months. This will include measurement of a range of novel factors involved in immune system activity including B lymphocyte activating factor (BAFF), a proliferating-inducing ligand (APRIL)

For peer review only - http://bmjopen.bmj.com/site/about/guidelines.xhtml

and B lymphocyte chemoattractant (CXCL13). This data will be analysed separately from the main trial.

Withdrawal

Participants have the right to withdraw from the trial at any time, including during the single infusion of RTX, without having to give a reason. The patient will still be asked to complete follow up. Sites will need to record the amount of RTX that was administered prior to the patients' withdrawal. Investigator sites should try to ascertain the reason for withdrawal and document this reason within the Case Report Form and participant's medical notes.

As this is a single dose administration it is not anticipated that the investigator would withdraw a patient from treatment (the infusion would be slowed down accordingly –see ***Intervention*** section). If the RTX infusion has to be stopped completely and cannot be recommenced then those subjects receiving less than 100mg RTX will be reviewed according to the protocol for safety reasons only. Should a participant withdraw from the trial then every effort will be made to obtain follow-up data, with the permission of the patient.

Participants who withdraw from the trial will not be replaced.

Sample Size

Formal justification to proceed to a larger randomised trial is based upon observing a minimum number achieving remission 2 years post RTX administration; this number is referred to as the critical number [43]. The critical number depends upon the desired and unacceptable remission rates, set at 40% and 20%, respectively, and the error levels set at 10% alpha (type I) and 20% beta (type II). The remission rate corresponding to the critical number will lie between 40% and 20% and will be specified and stored separately in the

For peer review only - http://bmjopen.bmj.com/site/about/guidelines.xhtml

Statistics TMF. If the true remission rate is 40% there is an 80% chance (power) of proceeding to a further trial; if the true remission rate is 20%, there is a small 10% (alpha) chance of proceeding to a further trial. With these parameters the target recruitment is 27 patients who will receive RTX. This is the smallest number of patients which satisfies the design criteria, assuming a 10% loss to follow-up.

Statistical Analysis

Patients will receive a single dose of RTX once they have fulfilled the inclusion and exclusion criteria. In the event that RTX is not well tolerated, only patients who were able to receive more than 100mg of RTX will be included in the statistical analysis.

Analysis of the Primary Outcome Measure

The number of patients in remission 2 years after RTX administration will be compared to the critical number. This is equivalent to a formal comparison of the hypotheses that the remission rate is greater than or equal to 40% as opposed to less than or equal to 20%. The primary outcome measure, remission rate, will be presented with a one sided 90% lower bound. If this phase II trial provides evidence that the true remission rate is plausibly 40% or more, 2 years after a single dose of RTX and a 12 month course of ATD, then this will indicate a likely effect of RTX on disease outcome and justify a randomised efficacy evaluation of this adjuvant RTX regimen.

Analysis of Secondary Outcome Measures

1. The distribution of TRAb titres will be compared between patients in and out of remission by graphical summary, numerical summary statistics (median and interquartile range) and a Mann-Whitney test.

For peer review only - http://bmjopen.bmj.com/site/about/guidelines.xhtml

2. Time to recovery of peripheral blood B cell lymphocyte numbers (CD 19+ cells) to above 70% of the patient's pre RTX level in relation to thyroid hormone status. This will be compared between patients in and out of remission by graphical summary, numerical summary statistics (median and interquartile range) and a Mann-Whitney test.
3. Cumulative dose of ATD (Carbimazole) in mg/kg in relation to thyroid hormone status. This will be compared between patients in and out of remission by graphical summary, numerical summary statistics (median and interquartile range) and a Mann-Whitney test.
4. We will assess the time taken for TSH concentrations to normalise (increase above 0.1mU/l) post RTX and describe biochemical thyroid status, as assessed by TSH, FT4 and FT3 concentration, in the period between cessation of ATD therapy at the end of year one and the end of the trial. Specifically we will plot the time taken for TSH to normalise in individual patients and determine the proportion of time that TSH, FT4 and FT3 concentrations are within the normal reference range in the period between ATD stopping (end of year 1) and trial end (end of year 2).
5. The frequency and nature of adverse events.

Criteria for the Premature Termination of the Trial

The criteria for stopping the trial will be if a patient dies from a RTX treatment related infection, or if 2 patients experience the same SUSAR which has severe or life-threatening consequences.

Safety reporting

We do not aim to collect adverse events such as minor trauma (scratches, cuts) in patients who are otherwise well. Adverse events will otherwise be classified according to whether they are:

For peer review only - http://bmjopen.bmj.com/site/about/guidelines.xhtml

1. Infections (minor = self-limiting infection such as the common cold, modest= non-self-limiting where an antibiotic is prescribed but the clinical course and recovery is not related to the underlying condition and its' treatment or severe where antibiotic therapy +/- other therapy is required with the infection related to drug effects on immune function).
2. Immune function related such as neutropaenia.
3. Related to the skin e.g. rashes.
4. Related to the musculo-skeletal system such as joint pain.
5. Related to gastro-intestinal upset.
6. Related to hepatic function.
7. Others

The trial will be stopped if a patient dies from a RTX treatment related infection or if 2 patients experience the same SUSAR which has severe or life-threatening consequences.

According to section 4.6 of the SmPC (Mabthera [the product name for RTX] 100mg and 500mg Concentrate for Solution for Infusion), RTX should not be administered to pregnant women. The SmPC states that "Rituximab should not be administered to pregnant women unless the possible benefit outweighs the potential risk". Due to the long retention time of RTX in B-cell depleted patients, women of childbearing potential need to use effective contraceptive methods during treatment and for 12 months following RTX therapy (in line with the SmPC for RTX). If a female participant becomes pregnant within 12 months of taking the RTX drug the details of the pregnancy needs to be reported to the Chief Investigator, Trial Manager and Sponsor within 24 hours of the site learning of its occurrence on the pregnancy reporting form.

For peer review only - http://bmjopen.bmj.com/site/about/guidelines.xhtml

If a patient or their partner (where the participant is male) becomes pregnant, within 12 months of taking RTX, the pregnancy must be reported as per the trial specific guidance document for pregnancy reporting and followed up to completion of pregnancy.

Patient and Public Involvement:

We were keen to involve patients including those with Graves' hyperthyroidism and their families in the design of this clinical trial. We therefore discussed the proposed trial with patients who had Graves' hyperthyroidism within our clinical service and liaised with the Young Person's Advisory Group (YPAG) in the North of England. We also discussed the trial with the British Thyroid Foundation (BTF) and it was clear from these discussions that improving the treatment options for young people with this condition was an important area. YPAG and a representative from the BTF helped to refine the trial protocol and the patient information sheets. A member of the BTF was part of the trial steering committee and the BTF published information regarding the trial on their website and facilitated recruitment by providing information to potential participants.

We intend to notify participants in writing regarding the trial outcome.

Ethics and Dissemination

The trial has received a favourable ethical opinion (North East - Tyne and Wear South Research Ethics committee, reference 16/NE/0253, EudraCT number 2016-000209-35).Trial oversight consists of Trial Steering Committee (TSC), Data Monitoring Committee (DMC) and Trial Management Group (TMG). The TSC comprises an Independent Chair and three other independent members including a statistician. The DMC consists of three independent members, an endocrinologist (Chair), immunologist and a statistician. The TMG will be

For peer review only - http://bmjopen.bmj.com/site/about/guidelines.xhtml

responsible for overseeing the progress of the trial. The day-to-day management of the trial will be co-ordinated by Newcastle Clinical Trials Unit who will also be responsible for informing sites regarding protocol modifications and updates. Data management will be undertaken in accordance with Newcastle University Clinical Trials Unit Standard Operating Procedures.

The results of this trial will be distributed widely at international endocrine meetings including the European Society for Paediatric Endocrinology annual meeting. The results will be published the peer-reviewed literature and distributed via patient support groups including the British Thyroid Foundation.

For peer review only

For peer review only - http://bmjopen.bmj.com/site/about/guidelines.xhtml

Discussion

Graves' disease remains a challenge for young people and their families with significant disadvantages associated with all standard treatment modalities. Most young people will ultimately be treated with thyroid hormone replacement which is not ideal from a practical, financial and quality of life perspective [44]. The management of Graves' hyperthyroidism is currently under review by NICE.

This is a proof of concept trial that may be the precursor to a phase III trial. The limitations of the trial design include the need to adopt a largely pragmatic approach to the selection of the Rituximab dose and the fact that an impact on disease course may not be detected by a 2 year trial. On the other hand, patients who remit by 2 years may still be at risk of relapse at a later stage. There is also a risk that patients will experience side-effects from ATD therapy that mean this has to be stopped. If so, then definitive treatment may be required before the impact of RTX on the disease course can be elucidated. There is also a need to be cautious about assuming that remission rates following a course of ATD in a 12 year old patient with Graves' hyperthyroidism are similar to those in someone who is 20 years of age. Remission rate may therefore reflect the age of trial participants as well as the impact of the treatment regimen.

We feel that there are three disadvantages from the patient's perspective when taking part in this trial. Firstly, the infusion involves a day spent in hospital. The second disadvantage is the potential risk of adverse events, notably infections, although the literature does not suggest significant issues in an otherwise healthy and immune competent group of subjects like young patients with Graves' hyperthyroidism who have received one or two doses of Rituximab. Finally there is the uncertainty about whether the intervention will impact on disease outcome favourably.

For peer review only - http://bmjopen.bmj.com/site/about/guidelines.xhtml

The number of clinic visits will be similar to patients with Graves' managed routinely and will help to ensure that patients biochemistry is monitored carefully.

ATD does not cure young Graves' patients in the short term and is only associated with resolution of the hyperthyroid state in a minority of patients after several years of treatment. Surgery and radioiodine remove or destroy the gland and hence are not a cure either. Rituximab provides the prospect of an earlier remission in patients with Graves' hyperthyroidism who are therefore less likely to experience the side-effects of ATD. Rituximab may cure patients who would otherwise not have been cured by ATD and it may reduce the likelihood of long-term hypothyroidism. Re-purposing rituximab for this indication would mean an immediately translatable therapy with a well-established and favourable side-effect profile, and a likely reduction in costs now that it is off-patent.

For peer review only

For peer review only - http://bmjopen.bmj.com/site/about/guidelines.xhtml

Trial status

This manuscript is based upon trial protocol version 4.0 dated 27th November 2017. The RiGD trial opened to recruitment in October 2016 and is due to close to recruitment in October 2018.

For peer review only

For peer review only - http://bmjopen.bmj.com/site/about/guidelines.xhtml

List of abbreviations

Abbreviation	Definition
AE	Adverse Event
ALT	Alanine transaminase
AR	Adverse Reaction
ATD	Antithyroid Drug
BTF	British Thyroid Foundation
CA	Competent Authority
CBZ	Carbimazole
CI	Chief Investigator
CRF	Case Report Form
CTA	Clinical Trial Authorisation
CTIMP	Clinical Trial of an Investigational Medicinal Product
DGH	District General Hospital
DLT	Dose Limiting Toxicity
DMC	Data Monitoring Committee
DSUR	Development Safety Update Report
eCRF	Electronic case report form
EudraCT	European Clinical Trials Database
EMA	European Medicines Agency
FT3	Free Tri-iodothyronine
FT4	Free Thyroxine
GCP	Good Clinical Practice
GD	Graves' Disease
GO	Graves' Orbitopathy
HBsAg	Hepatitis 'B' surface antigen
HB Core	Hepatitis 'B' Core
HCV	Hepatitis 'C' virus
HIV	Human Immunodeficiency Virus
HRA	Health Research Authority
HTA	Human Tissue Authority
HTAct	Human Tissue Act
IB	Investigator Brochure
ICF	Informed Consent Form
ICH	International Conference on Harmonisation
IMP	Investigational Medicinal Product
ISF	Investigator Site File
ISRCTN	International Standard Randomised Controlled Trials Number
MA	Marketing Authorisation
MHRA	Medicines and Healthcare products Regulatory Agency
MP	Methylprednisolone
NCTU	Newcastle Clinical Trials Unit
NHS	National Health Service
PI	Principal Investigator
PIS	Participant Information Sheet
PK	Pharmacokinetic
PTU	Propylthiouracil

For peer review only - http://bmjopen.bmj.com/site/about/guidelines.xhtml

QA	Quality Assurance
QC	Quality Control
QP	Qualified Person
R&D	Research & Development
REC	Research Ethics Committee
RSI	Reference Safety Information
RI	Radio-iodine
RTX	Rituximab (Mabthera ®)
SAE	Serious Adverse Event
SAR	Serious Adverse Reaction
SDV	Source Data Verification
SLE	Systemic Lupus Erythematosus
SOP	Standard Operating Procedure
SmPC	Summary of Product Characteristics
SSI	Site Specific Information
SUSAR	Suspected Unexpected Serious Adverse Reaction
TBII	Thyroid Binding Inhibiting Immunoglobulin
TMF	Trial Master File
TMG	Trial Management Group
TPO	Thyroid Peroxidase
TRAb	Thyroid receptor antibody
TSC	Trial Steering Committee
TSH	Thyroid Stimulating Hormone
TSHR	Thyroid Stimulating Hormone Receptor

For peer review only - http://bmjopen.bmj.com/site/about/guidelines.xhtml

Declarations

Ethics approval and consent to participate

REC approval was the 15th of September 2016. The first patient was consented on the 4th November 2016.

Consent for publication

Not applicable

Availability of data and materials

Not applicable

Competing interests

The authors declare that they have no competing interests

Funding

The trial is funded by a grant from the Medical Research Council Biomedical Catalyst: Developmental Pathway Funding Scheme (DPFS).

Authors' contributions

SP and TC had the original idea for the trial. The trial protocol was developed by TC with substantial input and revision by SP, MC, DH, MA and AMH. LH and AD also made changes to the protocol at various stages of development. Comments from all other co-authors helped to refine the protocol during the final stages leading up to the start of patient recruitment (AA, TB, KB, PD, JK, NZ). The trial protocol manuscript was primarily drafted by MC and TC with substantial input from DH and MA. All other co-authors approved the final version of the manuscript. All trial co-applicants and investigators who recruit patients to the trial will be eligible for co-authorship.

For peer review only - http://bmjopen.bmj.com/site/about/guidelines.xhtml

Acknowledgements

We would like to acknowledge the guidance provided by the local young person's advisory group and by the British Thyroid Foundation when the trial documentation was being developed. We would also like to acknowledge the ongoing support provided by the clinical and research teams from the endocrine units in Cardiff, Doncaster, Leeds and Southampton who opened as participating sites after the trial commenced.

References

1 Flynn RWV, MacDonald TM, Morris AD, Jung RT, Leese GP. The thyroid epidemiology, audit, and research study: Thyroid dysfunction in the general population. J Clin Endocr Metab. 2004;89:3879-3884.

2 McDonald DO, Pearce SHS. Thyroid peroxidase forms thionamide-sensitive homodimers: relevance for immunomodulation of thyroid autoimmunity. J Mol Med-Jmm. 2009;87:971-980.

3 Rivkees SA, Stephenson K, Dinauer C. Adverse Events Associated with Methimazole Therapy of Graves' Disease in Children. International Journal of Pediatric Endocrinology. 2010;2010:176970.

4 Rivkees SA, Sklar C, Freemark M. The Management of Graves' Disease in Children, with Special Emphasis on Radioiodine Treatment. The Journal of Clinical Endocrinology & Metabolism. 1998;83:3767-3776.

5 Lazar L, Kalter-Leibovici O, Pertzelan A, Weintrob N, Josefsberg Z, Phillip M. Thyrotoxicosis in Prepubertal Children Compared with Pubertal and Postpubertal Patients. The Journal of Clinical Endocrinology & Metabolism. 2000;85:3678-3682.

6 Glaser NS, Styne DM, Organization of Pediatric Endocrinologists of Northern California Collaborative Graves' Disease Study G. Predicting the likelihood of remission in children with Graves' disease: a prospective, multicenter study. Pediatrics. 2008;121:e481-488.

7 Cheetham T, Bliss R. Treatment options in the young patient with Graves' disease. Clinical endocrinology. 2016;85:161-164.

For peer review only

For peer review only - http://bmjopen.bmj.com/site/about/guidelines.xhtml

8 Franklyn JA, Boelaert K. Thyrotoxicosis. The Lancet. 2012;379:1155-1166.

9 Leandro MJ, Cambridge G, Ehrenstein MR, Edwards JC. Reconstitution of peripheral blood B cells after depletion with rituximab in patients with rheumatoid arthritis. Arthritis Rheum. 2006;54.

10 Boross P, Leusen JHW. Mechanisms of action of CD20 antibodies. Am J Cancer Res. 2012;2:676-690.

11 Edwards JCW, Szczepański L, Szechiński J, Filipowicz-Sosnowska A, Emery P, Close DR, Stevens RM, Shaw T. Efficacy of B-Cell–Targeted Therapy with Rituximab in Patients with Rheumatoid Arthritis. New England Journal of Medicine. 2004;350:2572-2581.

12 Jansson AF, Sengler C, Kuemmerle-Deschner J, Gruhn B, Kranz AB, Lehmann H, Kleinert D, Pape L, Girschick HJ, Foeldvari I, *et al.* B cell depletion for autoimmune diseases in paediatric patients. Clinical Rheumatology. 2011;30:87-97.

13 Alexeeva EI, Valieva SI, Bzarova TM, Semikina EL, Isaeva KB, Lisitsyn AO, Denisova RV, Chistyakova EG. Efficacy and safety of repeat courses of rituximab treatment in patients with severe refractory juvenile idiopathic arthritis. Clin Rheumatol. 2011;30:1163-1172.

14 Kavcic M, Fisher BT, Seif AE, Li Y, Huang YS, Walker D, Aplenc R. Leveraging administrative data to monitor rituximab use in 2875 patients at 42 freestanding children's hospitals across the United States. The Journal of pediatrics. 2013;162:1252-1258, 1258.e1251.

15 Lippe BM, Landaw EM, Kaplan SA. Hyperthyroidism in children treated with long term medical therapy: twenty-five percent remission every two years. The Journal of clinical endocrinology and metabolism. 1987;64:1241-1245.

16 Rheumatology BSfPaA. Clinical Guidelines. 2017 [cited 7 November 2017]. Available from: http://80.87.12.43/bspar/guidelines/default.aspx

17 Hauser SL, Waubant E, Arnold DL, Vollmer T, Antel J, Fox RJ, Bar-Or A, Panzara M, Sarkar N, Agarwal S, *et al.* B-cell depletion with Rituximab in relapsing-remitting multiple sclerosis. New England Journal of Medicine. 2008;358:676-688.

For peer review only

For peer review only - http://bmjopen.bmj.com/site/about/guidelines.xhtml

18 Chamberlain N, Massad C, Oe T, Cantaert T, Herold KC, Meffre E, Pathway TDT. Rituximab does not reset defective early B cell tolerance checkpoints. Journal of Clinical Investigation. 2016;126:282-287.

19 Kamei K, Ishikura K, Sako M, Aya K, Tanaka R, Nozu K, Kaito H, Nakanishi K, Ohtomo Y, Miura K*, et al.* Long-term outcome of childhood-onset complicated nephrotic syndrome after a multicenter, double-blind, randomized, placebo-controlled trial of rituximab. Pediatr Nephrol. 2017;32:2071-2078.

20 Oved JH, Lee CSY, Bussel JB. Treatment of Children with Persistent and Chronic Idiopathic Thrombocytopenic Purpura: 4 Infusions of Rituximab and Three 4-Day Cycles of Dexamethasone. J Pediatr-Us. 2017;191:225-231.

21 Tak PP, Rigby W, Rubbert-Roth A, Peterfy C, van Vollenhoven RF, Stohl W, Healy E, Hessey E, Reynard M, Shaw T. Sustained inhibition of progressive joint damage with rituximab plus methotrexate in early active rheumatoid arthritis: 2-year results from the randomised controlled trial IMAGE. Annals of the Rheumatic Diseases. 2012;71:351-357.

22 Verstappen GM, Kroese FGM, Meiners PM, Corneth OB, Huitema MG, Haacke EA, van der Vegt B, Arends S, Vissink A, Bootsma H*, et al.* B Cell Depletion Therapy Normalizes Circulating Follicular Th Cells in Primary Sjogren Syndrome. J Rheumatol. 2017;44.

23 Ohye H, Minagawa A, Noh JY, Mukasa K, Kunii Y, Watanabe N, Matsumoto M, Suzuki M, Yoshihara A, Ito K*, et al.* Antithyroid drug treatment for graves' disease in children: a long-term retrospective study at a single institution. Thyroid : official journal of the American Thyroid Association. 2014;24:200-207.

24 El Fassi D, Nielsen CH, Bonnema SJ, Hasselbalch HC, Hegedus L. B lymphocyte depletion with the monoclonal antibody rituximab in Graves' disease: a controlled pilot study. The Journal of clinical endocrinology and metabolism. 2007;92:1769-1772.

25 Salvi M, Vannucchi G, Campi I, Rossi S, Bonara P, Sbrozzi F, Guastella C, Avignone S, Pirola G, Ratiglia R*, et al.* Efficacy of rituximab treatment for thyroid-associated ophthalmopathy as a result of intraorbital B-cell depletion in one patient unresponsive to steroid immunosuppression. European journal of endocrinology. 2006;154:511-517.

26 El Fassi D, Nielsen CH, Hasselbalch HC, Hegedus L. Treatment-resistant severe, active Graves' ophthalmopathy successfully treated with B lymphocyte depletion. Thyroid : official journal of the American Thyroid Association. 2006;16:709-710.

For peer review only - http://bmjopen.bmj.com/site/about/guidelines.xhtml

27 Salvi M, Vannucchi G, Campi I, Curro N, Dazzi D, Simonetta S, Bonara P, Rossi S, Sina C, Guastella C, *et al.* Treatment of Graves' disease and associated ophthalmopathy with the anti-CD20 monoclonal antibody rituximab: an open study. European journal of endocrinology. 2007;156:33-40.

28 Heemstra KA, Toes RE, Sepers J, Pereira AM, Corssmit EP, Huizinga TW, Romijn JA, Smit JW. Rituximab in relapsing Graves' disease, a phase II study. European journal of endocrinology. 2008;159:609-615.

29 Mitchell AL, Gan EH, Morris M, Johnson K, Neoh C, Dickinson AJ, Perros P, Pearce SH. The effect of B cell depletion therapy on anti-TSH receptor antibodies and clinical outcome in glucocorticoid-refractory Graves' orbitopathy. Clinical endocrinology. 2013;79:437-442.

30 Salvi M, Vannucchi G, Curro N, Campi I, Covelli D, Dazzi D, Simonetta S, Guastella C, Pignataro L, Avignone S, *et al.* Efficacy of B-cell targeted therapy with rituximab in patients with active moderate to severe Graves' orbitopathy: a randomized controlled study. The Journal of clinical endocrinology and metabolism. 2015;100:422-431.

31 Wang CJ, Ning QY, Jin K, Xie JJ, Ye J. Does rituximab improve clinical outcomes of patients with thyroid-associated ophthalmopathy? A systematic review and meta-analysis. Bmc Ophthalmol. 2018;18.

32 Stan MN, Garrity JA, Leon BGC, Prabin T, Bradley EA, Bahn RS. Randomized Controlled Trial of Rituximab in Patients With Graves' Orbitopathy. J Clin Endocr Metab. 2015;100:432-441.

33 Stan MN, Salvi M. MANAGEMENT OF ENDOCRINE DISEASE Rituximab therapy for Graves' orbitopathy - lessons from randomized control trials. European journal of endocrinology. 2017;176:R101-R109.

34 Iijima K, Sako M, Nozu K, Mori R, Tuchida N, Kamei K, Miura K, Aya K, Nakanishi K, Ohtomo Y, *et al.* Rituximab for childhood-onset, complicated, frequently relapsing nephrotic syndrome or steroid-dependent nephrotic syndrome: a multicentre, double-blind, randomised, placebo-controlled trial. Lancet. 2014;384:1273-1281.

35 Kaplan B, Kopyltsova Y, Khokhar A, Lam F, Bonagura V. Rituximab and immune deficiency: case series and review of the literature. The journal of allergy and clinical immunology In practice. 2014;2:594-600.

For peer review only - http://bmjopen.bmj.com/site/about/guidelines.xhtml

36 Abraham P, Avenell A, McGeoch SC, Clark LF, Bevan JS. Antithyroid drug regimen for treating Graves' hyperthyroidism. The Cochrane database of systematic reviews. 2010:Cd003420.

37 McGregor AM, Petersen MM, McLachlan SM, Rooke P, Smith BR, Hall R. Carbimazole and the autoimmune response in Graves' disease. N Engl J Med. 1980;303:302-307.

38 Marcocci C, Chiovato L, Mariotti S, Pinchera A. Changes of circulating thyroid autoantibody levels during and after the therapy with methimazole in patients with Graves' disease. Journal of endocrinological investigation. 1982;5:13-19.

39 McDonald DO, Pearce SH. Thyroid peroxidase forms thionamide-sensitive homodimers: relevance for immunomodulation of thyroid autoimmunity. Journal of molecular medicine (Berlin, Germany). 2009;87:971-980.

40 Humar M, Dohrmann H, Stein P, Andriopoulos N, Goebel U, Roesslein M, Schmidt R, Schwer CI, Loop T, Geiger KK*, et al.* Thionamides inhibit the transcription factor nuclear factor-kappaB by suppression of Rac1 and inhibitor of kappaB kinase alpha. The Journal of pharmacology and experimental therapeutics. 2008;324:1037-1044.

41 Hamburger JI. Management of hyperthyroidism in children and adolescents. The Journal of clinical endocrinology and metabolism. 1985;60:1019-1024.

42 Wood LC, Ingbar SH. Hypothyroidism as a Late Sequela in Patients with Graves' Disease Treated with Antithyroid Agents. Journal of Clinical Investigation. 1979;64:1429-1436.

43 A'Hern RP. Sample size tables for exact single-stage phase II designs. Statistics in medicine. 2001;20:859-866.

44 Saravanan P, Chau WF, Roberts N, Vedhara K, Greenwood R, Dayan CM. Psychological well-being in patients on 'adequate' doses of L-thyroxine: results of a large, controlled community-based questionnaire study. Clinical endocrinology. 2002;57:577-585.

For peer review only

For peer review only - http://bmjopen.bmj.com/site/about/guidelines.xhtml

Appendices

Appendix 1 a Patient information sheet children

Appendix 1b Patient information sheet

Appendix 1c Assent form

Appendix 1d Consent form

For peer review only

For peer review only - http://bmjopen.bmj.com/site/about/guidelines.xhtml

RiGD

To be printed on Hospital headed paper

PARTICIPANT INFORMATION SHEET FOR CHILDREN

Short title: Rituximab in Graves' Disease

Study Question?

Will a new medicine called Rituximab help to make the thyroid gland work normally.

Brief Summary

Current standard NHS treatment for patients	Treatment you will get if you take part in this study.
1. You will see your doctor around 10 times over 2 years.	1. You will see your doctor 15 times over 2 years.
2. You will take anti-thyroid medicine for 2 years. (Once patients stop taking this medicine the disease will usually come back in 3 out of 4 patients)	2. You will take anti-thyroid medicine for 1 year and then be followed up in clinic for the next year.
3. If the disease comes back the options include surgery (removing your thyroid gland) or Radioiodine treatment (radioactive iodine that is taken up by the thyroid and destroys it) or returning to anti-thyroid medicine. After surgery or radioiodine treatment, patients usually take thyroid hormone replacement therapy for life.	3. You will get one dose of Rituximab treatment using a tube in a vein with a drip attached. This treatment may help to reduce the chances of the disease coming back when you stop the anti-thyroid medicine after 1 year. If your thyroid gland is still not working normally after the first year of treatment with us, we will be able to detect this. If this happens we will restart your anti-thyroid medicine. After the first year of treatment is finished, it is possible that your thyroid problem might come back. If this happens please let us know straight away and we will restart anti-thyroid medicine so you do not feel poorly.

[illegible]

You have been found to have [illegible] thyroid gland in the neck makes too much thyroid hormone. This can make you feel unwell.

Why me?

You have been picked [illegible]. You can help us find out if a new medicine called Rituximab will help to make the thyroid gland work

For peer review only - http://bmjopen.bmj.com/site/about/guidelines.xhtml

To be printed on Hospital headed paper

normally. The Rituximab medicine will be given together with a one year course of the normal treatment for this condition. Unfortunately the normal treatment (anti-thyroid drug usually taken as tablets that you swallow) does not usually make the thyroid gland work normally by itself.

We are asking 27 young people between 12 and 20 years old to take part in this study.

Do I have to take part?

No, you do not. It is up to you. We would first like you to read this information sheet with your mum, dad or carer and if you would like to take part we would then like you to confirm this by writing your name on a form.

We will also ask your mum, dad or carer to write down that they are happy for you to take part as well.

Remember, [illegible] just tell us.

What will happen?

[illegible], you would normally see your doctor about 10 times over 2 years. If you take part in the study we would like to see you 15 times over the next 2 years. If you have [illegible] ou would normally get a blood sample taken when you see your doctor. We would like to take a little extra blood to make sure that you are OK and to see if the new medicine is working and to do new tests to see how your immune system is working. If you are a girl, we will need to take some of your urine, 2 times during the study. We check your urine to make sure you are not pregnant. This is something we have to do in every female that takes part, irrespective of how old they are. Both the girls and boys that decide to take part in this study will need to use contraception if there is chance that you might get pregnant or have a baby. This is because it might not be good for the baby if you get pregnant in the 12 months after receiving the Rituximab medicine. Information on the different methods of contraception available can be found on the NHS webpage http://www.nhs.uk/Conditions/contraception-guide/Pages/what-is-contraception.aspx or call the national sexual health line on 0300 123 7123. You can also talk to the study team if you have any questions.

What happens when the study stops?

We will collect the information from the young people taking part and we will then decide if we need to have a bigger study. A bigger study will help to tell us for certain if Rituximab should be used in [illegible]

What if there is a problem?

Your mum, dad or carer will be able to talk to someone who will be able to tell them what they need to do about it.

[illegible]

Just tell your mum, dad, carer or doctor. They will not be cross with you. You will still be looked after when you are visiting hospital.

For peer review only - http://bmjopen.bmj.com/site/about/guidelines.xhtml

To be printed on Hospital headed paper

What if I wish to complain about the study?

If you have a concern about any aspect of this study or if you want to complain, you, or your mum, dad or carer can talk to your doctor _____ or nurse _____ at this hospital.

Will anyone else know [illegible]

The people in our research team will know you are taking part. The doctor looking after you while you are in hospital will also know and we will also inform your family doctor or GP. No one else will know because we will not use your name or address.

What happens to what the researchers find out?

When we collect your information we will make sure it is stored in a safe place and only the people doing the research study can look at it.
We will use the information to work out how best to treat [illegible] Disease. The Newcastle Clinical Trials Unit would like to receive a copy of your consent form for safety purposes. This will be destroyed once it has been reviewed.

How can I find out more about this study?

Your mum, dad, carer or other grown-up you trust may be able to answer some of your questions. The doctors and nurses looking after you can also help you all to find out more about the study.

Rituximab Treatment

Your nurse or doctor will give you Rituximab once using a tube in a vein with a drip attached. Local anaesthetic cream will be offered before the tube is put in place or before any blood tests so that it doesn't hurt. The drip will have a pump at the other end that will give you Rituximab over about 3 to 4 hours.

What could it do to me? Is it definitely safe?

Rituximab has been used to treat other diseases in lots of young and old people for the past 18 years. Half of the patients may feel a bit hot or cold, or sick or itchy for a short time but we will give you medicine to help prevent this. If you do feel poorly during the treatment then we will stop giving you Rituximab for a few minutes. When you feel better again we will give you Rituximab more slowly. You will be checked carefully as you get Rituximab to make sure you are feeling ok. There is a very small possibility that you might need antibiotic medicine to help you fight infections at some point in the next year. Although there is no guarantee that Rituximab and Anti Thyroid Drug (ATD) treatment will work for you, We need to do this study to work out how likely it is that this approach will benefit young people.

Thank you for taking time to read this □

Please ask any questions if you need to.

For peer review only - http://bmjopen.bmj.com/site/about/guidelines.xhtml

To be printed on local hospital headed paper

Adjuvant rituximab – a potential treatment for the young patient with Graves' hyperthyroidism.

Short title: **R**ituximab **i**n **G**raves' **D**isease

PATIENT INFORMATION LEAFLET

Invitation

You are being invited to take part in a research study. Please read the following information to help you decide if you want to take part. We would like you to understand why we are doing this research and what it means for you. You do not need to make a decision straight away, so please feel free to talk to others about the study if you wish. Please ask us if there is anything that is not clear or if you want to know more.

Please remember that you do not have to take part and your normal healthcare will not be affected in any way, whatever you decide.

(Brief summary to see if you want to continue to read)

- You have been asked to take part because you have been diagnosed with Graves' hyperthyroidism (also known as Graves' disease), which means you have an overactive thyroid gland. We are looking at a possible new treatment for Graves' disease using a medicine called Rituximab.

- The current standard treatment for Graves' disease involves;
  - Taking Anti Thyroid Drugs (ATD) for around 2 years and seeing your doctor for a check-up about 10 times over the same period of time. The Graves' disease will come back once you have stopped taking ATD in about 3 out of every 4 patients.
  - If ATD treatment does not work and the Graves' disease comes back then the other current treatment options (apart from returning to the ATD once again) are;
    1.) Surgery (removing your thyroid gland)
    2.) Radioiodine treatment. (Radioactive iodine is taken up by the thyroid, and destroys the cells in the thyroid gland).

For peer review only - http://bmjopen.bmj.com/site/about/guidelines.xhtml

To be printed on local hospital headed paper

- After surgery or radioiodine treatment, patients usually need to take thyroid hormone replacement for the rest of their life. Thyroid hormone replacement medicine is needed to replace the hormones which are missing after removal of the thyroid gland by surgery or after destruction by radioiodine treatment.

Why is a trial needed?

A trial is needed because the potential new treatment of Graves' disease that uses a combination of Rituximab and a short course of anti-thyroid drugs has not been studied before. Rituximab is given into a vein with a drip (an intravenous infusion) on one occasion and the anti-thyroid drugs are the tablets that are normally taken in this condition by mouth (orally). We do not know if this new treatment combination is more likely to restore your thyroid gland function to normal. The idea behind the study is that Rituximab might slow down or stop the the body's immune system from making your thyroid gland over-active. We would like to find out if the single dose of Rituximab, when taken together with a 1 year course of anti-thyroid drug, will increase the likelihood of the thyroid gland functioning normally when anti-thyroid drug treatment is stopped. This trial will recruit 27 young people, aged 12-20 years, who have recently been diagnosed with Graves' disease and who have only been on anti-thyroid drugs for 6 weeks at the most.

Standard or usual NHS care would mean that you see your thyroid doctor about 10 times over 2 years. If you take part in this trial we would like to see you 15 times over 2 years.

Please continue to read the following information to see if you would like to take part.

What is Rituximab?

Patients with Graves' Disease have too many abnormal B cells that are part of the body's immune system. Rituximab targets abnormal and normal B-cells. Once Rituximab treatment is over the normal B-cells will recover but we hope that the abnormal ones will not be replaced.

What would taking part involve?

In total we will need to see you 15 times in 2 years.

For peer review only - http://bmjopen.bmj.com/site/about/guidelines.xhtml

RiGD

To be printed on local hospital headed paper

Introductory visit (Total time approx. 30 minutes)

We will discuss the study in full and you can ask any questions you might have about your diagnosis. You would then go away and think about it for a few days.

Screening and Consent visit (total time 1 hour)

We will check that you understand the study and are happy to take part. We will then ask you to sign a consent form and we will take a blood sample (less than 1 teaspoon in amount) to check for a viral infection of the liver (hepatitis) and also (only for female patients) to undertake a pregnancy test. This is something we have to do for every female that takes part, irrespective of what age they are. If either of these tests are positive we will let you know. This means that you would not be eligible to take part in the study. You would then return to standard NHS care. Some units may routinely screen for other infections before administering Rituximab.

Rituximab Treatment visit (Total time 6 hours).

This visit will only happen if the screening visit bloods confirm that there is no evidence of hepatitis infection and that you are not pregnant (female patients). You will then have a thin plastic cannula (tube) inserted into your arm vein and 10mls of blood (less than 3 teaspoons) will be taken. Local anaesthetic cream will be offered before the tube is put in place or before any blood tests. A member of the study team will then give you Rituximab into a vein with a drip (an intravenous infusion). They will give the Rituximab treatment using a pump, which will give you the medicine over a 3 to 4 hours. You will be monitored in hospital for a further 2 hours following the treatment before you can go home. After this visit and subsequent visits a member of the team will call you to tell you what dose of anti-thyroid drugs you need to take.

For the next 12 months (Visit times 30 minutes approx.)

During the first 12 months of the study you will be seen in hospital to review the dose of your anti-thyroid drug. We will check to see how you are feeling and record if you have noticed any side effects. We will then take up to 8 mls of blood, less than 2 teaspoons of blood to make sure that you are OK. After each

For peer review only - http://bmjopen.bmj.com/site/about/guidelines.xhtml

To be printed on local hospital headed paper

visit a member of the study team will call you to tell you what dose of anti-thyroid drug you need to take.

1 year after Rituximab Treatment (Visit time 30 minutes approx.).

You will be asked to stop taking anti-thyroid drugs at this visit. We will check to see how you are feeling and record if you have noticed any side effects. We will need to take 8mls of blood (less than 3 teaspoons) to make sure you are OK and for an investigation into newly discovered markers of the body's immune system works. Female patients will need to give a urine sample to rule out pregnancy.

The final 12 months (Each visit time lasts half an hour approximately.)

When you come to the hospital we will check to see what medications you are taking. We will check to see how you are feeling. If the hyperthyroidism returns then we may detect this before you develop symptoms. If you start to feel as though the hyperthyroidism is coming back between clinic visits then you can let us know straight away so you don't become poorly. We will need to take 12mls of blood (less than 3 teaspoons) to make sure that you are OK and to see if the Graves' disease has returned. Once the 2 years are over you will then return to normal NHS endocrinology care.

SUMMARY of main study visits

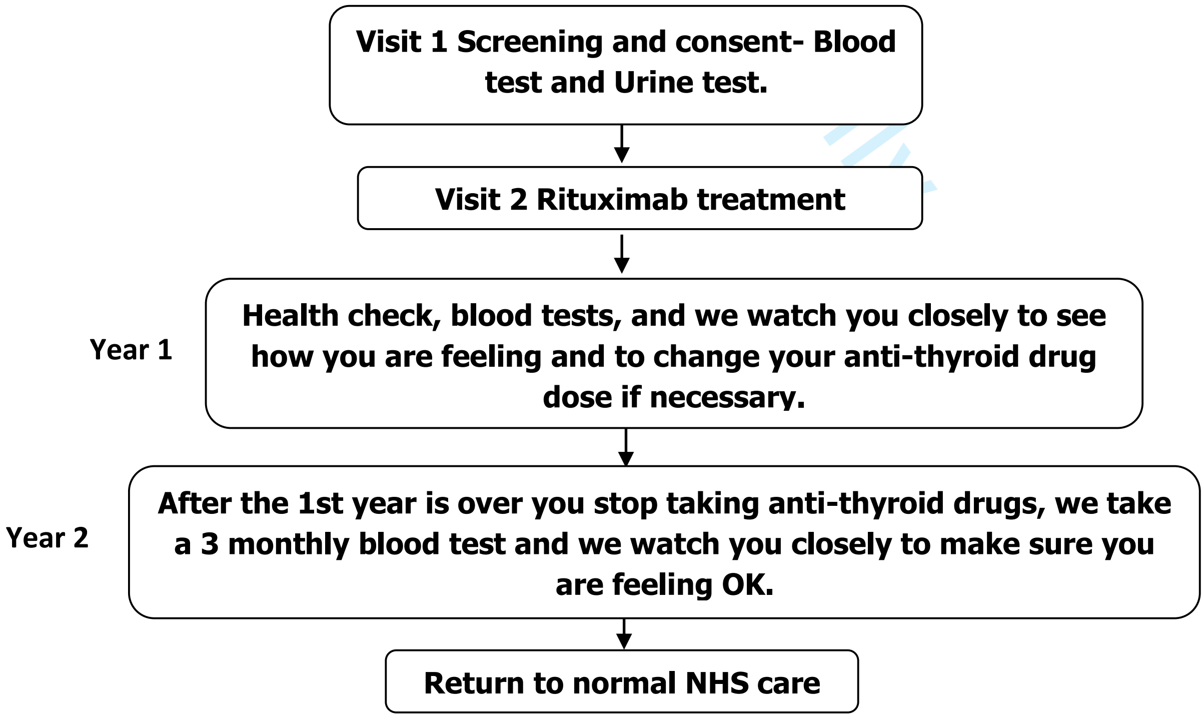

For peer review only - http://bmjopen.bmj.com/site/about/guidelines.xhtml

RiGD

To be printed on local hospital headed paper

What is it like to receive Rituximab?

Rituximab has been in use for over 18 years, with more than a million people treated world-wide – including many young people. It has been used successfully in many people with other autoimmune conditions (Graves' disease is also an autoimmune condition). You will receive one dose of the Rituximab. Around half of the people receiving a Rituximab infusion will feel some effects from having the drug. These can include, feeling hot or cold, shivering, feeling sick, or itchiness. We use other medication (such as paracetamol) to prevent this but if it does happen then the treatment will be stopped for a few minutes, until you feel better, and then restarted at a slower rate. You will be monitored carefully during the treatment and watched carefully if you feel poorly.

What happens after the treatment?

Following the treatment, you will go home with your anti-thyroid tablets. We advise for the first 6 months following treatment that patients do not receive any live vaccines. For the first 12 months we will want to keep a close eye on you to check you are well and, if necessary, to adjust the dose of the anti-thyroid drug. We expect that your dose of anti-thyroid tablets will be reduced during the first year of the study. For the final 12 months of the study we will see you every 3 months to check you are well and to see if the Graves' disease has returned.

What do I do next if I am considering taking part in the study?

You may have further questions to ask, and you may wish to discuss it with family members, friends, your own family doctor or hospital specialist. Please ask the study team anything you like about this trial. Remember that if you do agree to participate, you can decide not to continue at any stage and that your treatment will not be affected in any way, whatever you decide about the study. Take your time, and tell your hospital consultant if you wish to go ahead.

For peer review only - http://bmjopen.bmj.com/site/about/guidelines.xhtml

RiGD

To be printed on local hospital headed paper

Your local contact people for the study are:

Contact Details of local PI:

Name:
Address:
Phone:
Email:

Contact details of local Research Nurse:

Name:
Address:
Phone:
Email:

What are the possible disadvantages and risks of taking part?

There is no guarantee that Rituximab and ATD treatment will work for you. We need to do this study to work out how likely it is that this approach will benefit patients.

As Rituximab is acting to alter your immune system, there is a small risk (1 in 50) that you might develop a serious infection, such as pneumonia, after the infusion. If this happens to you then you may have to be hospitalised to receive antibiotic treatment. However, most people have Rituximab without any infection occurring as a result.

You will be asked to come to the research unit 15 times during the 2 year study. This is a considerable commitment in terms of your time and you will need to think carefully about whether you can fit in this number of visits.

Can I take part if I (or my partner) am pregnant or are planning on becoming pregnant?

We cannot give Rituximab to any females that are pregnant or planning on becoming pregnant during the study, because of the unknown risks to unborn babies. We also cannot give Rituximab to any males that are planning a pregnancy with their partner during the study.

All males and females participating in the study must agree to use effective forms of contraception for at least 12 months following the Rituximab treatment,

For peer review only - http://bmjopen.bmj.com/site/about/guidelines.xhtml

To be printed on local hospital headed paper

(The study team will discuss effective forms of contraception with you.) Information on the different methods of contraception available can be found on the NHS webpage http://www.nhs.uk/Conditions/contraception-guide/Pages/what-is-contraception.aspx or call the national sexual health line on 0300 123 7123. Rituximab might stay in your body for up to 12 months after the infusion.

If you or your partner becomes pregnant during the study you must tell the research team immediately.

Will I be paid for being in the trial?

No, you will not be paid for being in the trial although we will reimburse all travel expenses.

What if there is a problem?

If you have a concern about any aspect of the study please contact your local doctor (see contact details above) or the doctor running this study, Dr Tim Cheetham (0191 282 9562 or ask for Dr Cheetham to be air-called via the Royal Victoria Infirmary Switchboard on 0191 233 6161) to discuss your concerns. If you are still unhappy and wish to complain formally and confidentially you can do this through the NHS complaints Procedure by speaking to a member of the PALS (Patient Advice and Liaison Service) on 0800 0320 202 or <site to localise with phone number and email address>

What will happen if I don't want to carry on with the study?

You can stop taking part in the study at any time and for any reason, without having to tell us why. We will keep any information and samples that you have given us so far for research. By signing the consent form you agree to this. If you do withdraw from the study, you will still need to be seen on a regular basis by a hospital team who are familiar with the normal management of Graves' disease.

Will my GP be told about my involvement in this study?

If you decide to take part in this study we will inform your GP. It will also be noted in your hospital medical records.

What will happen to any samples that I give?

For peer review only - http://bmjopen.bmj.com/site/about/guidelines.xhtml

To be printed on local hospital headed paper

You will have given us 15 blood samples for the study (normal NHS care would involve about 10). The serum from your blood for 3 of these samples will be sent to a laboratory at Newcastle upon Tyne. The team there will use the blood samples to look at the level of antibodies that cause Graves' disease and the levels of thyroid hormone and for an investigation into newly discovered markers of the way the body's immune system works. Two samples will be stored for about 5 years just in case we need to test for other antibodies in future.

Will my personal details be kept confidential?

All personal details will be kept confidential. The study data in your medical notes will be looked at by people directly involved in the study, as well as by people who are monitoring and auditing the study. This may include the Newcastle Clinical Trials Unit, regulatory authorities or the hospital to make sure the study is being run correctly. We will need to take your contact details to get in touch with you by telephone or via email to give you your new ATD dose after study visits 1-9. We may also need to contact you regarding long term thyroid status and longer term well-being. Any personal information will be held securely and safely for the time of the study at your hospital. When the study ends this information needs to be kept (archived) for at least 5 years. This allows any queries about the conduct of the study to be resolved. This archived information will be kept very securely by the hospital to protect you. Any data collected during the study held by the central study team at Newcastle will be anonymised and will only have your study ID included. The Newcastle Clinical Trials Unit would like to receive a copy of your consent form for safety purposes. This will be destroyed once it has been reviewed.

What will happen to the results of the research study?

The results of the study will be published in scientific journals. You will not be named in any publication. We would also like to send you a newsletter with a summary of our results. Please let the research team know if you do not want to receive the newsletter.

Who is organising and funding the research?

This study has been funded by the Medical Research Council, United Kingdom. This study is sponsored and indemnified by the Newcastle Upon Tyne NHS

For peer review only - http://bmjopen.bmj.com/site/about/guidelines.xhtml

RiGD

To be printed on local hospital headed paper

Foundation Trust and Newcastle University. The Newcastle Clinical Trials Unit is organising the study.

Who has reviewed the study?

All research in the NHS is looked by an independent group of people called a NHS Research Ethics Committee (REC). This is to protect your interests. This study has been reviewed and given a favourable opinion by North East – Tyne & Wear South Research Ethics Committee and the NHS Health Research Authority.

What if relevant new information becomes available?

The study team will ensure the patients are receiving the most appropriate and up to date medical care they require.

How have patients and the public been involved in this study?

The Young Persons Advisory Group (YPAG) based in Newcastle upon Tyne have reviewed this Patient Information Leaflet and consent form. The Founding Director and Secretary to the Trustees of the British Thyroid Foundation has also reviewed this Patient Information Leaflet and consent form.

Thank you for taking time to read this leaflet.

For peer review only

For peer review only - http://bmjopen.bmj.com/site/about/guidelines.xhtml

RiGD

To be printed on Hospital headed paper

Young Person aged (12-15 years) Participant Assent Form v4.0

Adjuvant rituximab – a potential treatment for the young patient with Graves' hyperthyroidism.

Short title: Rituximab in Graves' Disease

Centre Number: ________________

Participant Number: ________________

Please INITIAL the boxes if you agree:

1	I have read and understood the Participant Information Sheet version _________ dated __________.	
2	I have had the chance to ask questions about the study and am happy with the answers given.	
3	I understand that I will need to give a blood sample when I come to hospital.	
4	I understand that some of the blood I give for this study will be used for new Exploratory analyses tests	
5	I understand that if I do not have to take part in the RiGD study, I know that I can stop at any time and I do not need to give a reason.	
6	I agree to take part in this study.	
7	I would like a copy or summary of the results to be sent to me when the study has finished.	
8	I agree to using the forms of contraception that have been explained to me if I am sexually active.	
9	I agree for a copy of this consent form to be sent securely to the Newcastle Clinical Trials Unit for safety purposes.	

To be answered by Female Participants only

10	I understand that I will need to provide 2 urine samples during the study to make sure that I am not pregnant. I understand that this is for safety purposes.	

Name of Child **Child to write Name here** **Date**

Name of person taking consent **Signature** **Date**

When completed 1 copy for participant, 1 for researcher site file, 1 (original) to be kept in medical notes and 1 copy to send securely to Newcastle Clinical Trials Unit

For peer review only - http://bmjopen.bmj.com/site/about/guidelines.xhtml

To be printed on Hospital headed paper

Adjuvant rituximab – a potential treatment for the young patient with Graves' hyperthyroidism. (16yrs +)

Short title: Rituximab in Graves' Disease

Participant Consent Form V4.0

Centre Number: ___________________ **Participant Number:** _____________

Please INITIAL the boxes if you agree:

1	I have read and understood the Participant Information Sheet version _____ dated ___________. I have had the opportunity to consider the information, to ask questions and am happy with the answers given.	
2	I understand that my participation is voluntary and that I am free to withdraw at any time without giving any reason, without my medical care or legal rights being affected.	
3	I consent to the collection of blood samples as part of this study. I understand that my samples will not be identifiable to me except to the study doctor and the direct research team.	
4	I understand that if I do withdraw and if I have already provided any of the 3 blood samples needed for the laboratory in Newcastle, these will still be used for the study and for exploratory analyses. I understand that no more information will be collec after I withdraw unless I give my permission.	
5	I understand that relevant sections of my medical notes and data collected during the study, may be looked by individuals from Newcastle University Clinical Trials Unit, from regulatory authorities or from the NHS Trust, where it is relevant to my taking part in this research. I give permission for these individuals to have access to my records.	
6	I understand that any personal information collected about me for the study will be kept confidential and not be made public. Information from the study will be published in medical journals and at research meetings. I understand that I will not be directly identified in the published results.	
7	I consent for the summary of the results to be sent to me when the study has finished.	
8	I agree for my name, address and contact telephone number and email address (if you have one) to be given to the study team. This is to allow the study team to contact me to inform me of how much anti-thyroid drug I need to take. The email address is ideally required to send communications about the study.	
9	I agree to my General Practitioner being informed about my taking part in this study.	
10	I agree to use the forms of contraception that have been explained to me (if I am sexually active).	
11	I understand that the information collected about me will be used to support other research in the future, and may be shared anonymously with other researchers.	
12	I agree to take part in the above study	
13	I give my permission for a copy of this consent form to be sent securely to the Newcastle Clinical Trials Unit for checking. This is for safety purposes.	

To be answered by Female Participants only

1	I understand that I will need to provide 2 urine samples during the study to make sure that I am not pregnant. I understand that this is for safety purposes.	

Name of participant **Signature** **Date**

Name of person taking consent **Signature** **Date**

When completed 1 copy for participant, 1 for researcher site file and 1 (original) to be kept in medical notes and 1 copy to send securely to Newcastle Clinical Trials Unit.

For peer review only
For peer review only - http://bmjopen.bmj.com/site/about/guidelines.xhtml

SPIRIT
STANDARD PROTOCOL ITEMS: RECOMMENDATIONS FOR INTERVENTIONAL TRIALS

SPIRIT 2013 Checklist: Recommended items to address in a clinical trial protocol and related documents*

Section/item	Item No	Description
**Administrative information**		
Title	1	Yes – page 1
Trial registration	2a	Yes – page 4
	2b	Throughout
Protocol version	3	Page 29: trial protocol version 4.0 dated 27th November 2017
Funding	4	MRC funding is noted on page 32
Roles and responsibilities	5a	Yes – page 2
	5b	Page 4
	5c	n/a
	5d	Outlined on page 1,2, 25 and 26.
**Introduction**		
Background and rationale	6a/b	Outlined on page 6 to 9
Objectives	7	Yes - Page 10
Trial design	8	Page 10 and 11
**Methods: Participants, interventions, and outcomes**		
Study setting	9	Yes - page 11
Eligibility criteria	10	Page 11 and 12
Interventions	11a	Yes – page 14 to 18
	11b	Yes – page 15
	11c	n/a

For peer review only

	11d	Surveillance schedule outlined on page 18
Outcomes	12	Outcomes on page 19
Participant timeline	13	Assessments / interventions outlined in table on page 18
Sample size	14	Explained on page 21 and 22
Recruitment	15	Screening, recruitment and consent strategies are on page 13.
**Methods: Assignment of interventions (for controlled trials)**		
Allocation:		
Sequence generation	16a	n/a
Allocation concealment mechanism	16b	n/a
Implementation	16c	Enrolment process is explained on page 13
Blinding (masking)	17a	n/a – not a blinded trial
	17b	n/a – not a blinded trial
**Methods: Data collection, management, and analysis**		
Data collection methods	18a	The data collection plan and involvement of the Trial management group and clinical trial unit is outlined on page 18 and 25
	18b	This issue is addressed on page 21
Data management	19	Data management will be undertaken in accordance with Newcastle University Clinical Trials Unit SOPs - https://www.ncl.ac.uk/nctu/activities/sop/library/, specifically: DM-010-01 Data Management; DM-011-01 Database Setup and Maintenance; and DM-012-01 Release of Data & Database Lock. (page 26).
Statistical methods	20a	Yes – page 22
	20b	Yes – page 22
	20c	Explained on page 22
**Methods: Monitoring**		
Data monitoring	21a	Makeup of DMC is on Page 25
	21b	Criteria for stopping trial explained on page 23

Harms	22	Safety reporting outlined on page 23
Auditing	23	Page 25 and 26
**Ethics and dissemination**		
Research ethics approval	24	Favourable ethical opinion explained on page 25
Protocol amendments	25	Page 26
Consent or assent	26a	Page 13
	26b	n/a
Confidentiality	27	In accordance with Newcastle University Clinical Trials Unit SOPs – see Data Management section 19.
Declaration of interests	28	Page 32 – no financial and other competing interests
Access to data	29	In accordance with Newcastle University Clinical Trials Unit SOPs.
Ancillary and post-trial care	30	Page 17
Dissemination policy	31a	Plans to disseminate results / inform participants are on page 25 and 26
	31b	Page 32
	31c	No plans as yet.
**Appendices**		
Informed consent materials	32	See appendices.
Biological specimens	33	Outlined in table 2, page 18

*It is strongly recommended that this checklist be read in conjunction with the SPIRIT 2013 Explanation & Elaboration for important clarification on the items. Amendments to the protocol should be tracked and dated. The SPIRIT checklist is copyrighted by the SPIRIT Group under the Creative Commons "Attribution-NonCommercial-NoDerivs 3.0 Unported" license.

For peer review only - http://bmjopen.bmj.com/site/about/guidelines.xhtml
